# Integrated transcriptomic and metabolomic profiles reveal the protective mechanism of modified Danggui Buxue decoction on radiation-induced leukopenia in mice

**DOI:** 10.3389/fphar.2023.1178724

**Published:** 2023-08-03

**Authors:** Wei Chen, Jiayun Xin, Xintong Wei, Qianqian Ding, Yunheng Shen, Xike Xu, Yanping Wei, Yanhui Lv, Jie Wang, Zhanhong Li, Weidong Zhang, Xianpeng Zu

**Affiliations:** ^1^ School of Pharmacy, Naval Medical University, Shanghai, China; ^2^ School of Pharmacy, Shandong University of Traditional Chinese Medicine, Jinan, China; ^3^ School of Pharmacy, Anhui University of Chinese Medicine, Hefei, China; ^4^ School of Pharmacy, Guangdong Pharmaceutical University, Guangzhou, China

**Keywords:** leukopenia, radiation, modified Danggui Buxue decoction (MDBD), transcriptomics, metabolomics

## Abstract

Leukopenia caused by radiation hinders the continuous treatment of cancers. Danggui Buxue Decoction (DBD) has been widely used in clinical owing to low toxicity and definite therapeutic effects to increase leukocytes. Meanwhile, icaritin (ICT) has also been proved to have the effect of boosting peripheral blood cells proliferation. However, there is no study to prove the efficacy of MDBD (Modified Danggui Buxue Decoction), a derivative herbal formula composed of DBD and ICT, in the treatment of radiation-induced leukopenia. In this study, we performed a model of 3.5 Gy whole-body radiation to induce leukopenia in mice. The results of pharmacodynamic studies demonstrated that MDBD could significantly increase the white blood cells in peripheral blood by improving the activity of bone marrow nuclear cells, reducing bone marrow damage, modulating spleen index, and regulating hematopoietic factors to alleviate leukopenia. We also analyzed the integrated results of metabolomics and transcriptomics and found that MDBD could relieve leukopenia and alleviate bone marrow damage by targeting steroid biosynthesis and IL-17 signaling pathway, in which the key genes are Jun, Cxcl2 and Egr1. Therefore, our study provides a basis for the effectiveness and compatibility in the combination of traditional Chinese medicine formula and small molecule drugs.

## 1 Introduction

Leukopenia is a commonly adverse effect associated with abnormal bone marrow hematopoiesis, especially during cancer-related radiotherapy (Shi C. et al., 2020). Leukocytes depletion is an unintended consequence of radiation toxicity, which results in the continuous decline of leukocytes in peripheral blood. Leukopenia, as the most common hematological toxicity caused by radiotherapy, has always been the main cause of treatment interruption (Franco et al., 2017), which affects the efficacy of radiotherapy and increases treatment time and economic burden. Leukopenia patients are taking a higher risk of infection, which has emerged as a serious threat to prevent patients from recovering (Kuo et al., 2014). Therefore, it is an essential step to reverse leukopenia in cancer treatment. Currently, the first-line drugs for leukopenia treatment include granulocyte-macrophage colony-stimulating factor (GM-CSF), granulocyte colony-stimulating factor (G-CSF), leucogen, vitamin B4, *etc.* (Bayne et al., 2012; Tian et al., 2019). However, numerous side effects appeared in their applications, such as bone tumors, fever, bone pain, and myalgia (Dai et al., 2010). Therefore, traditional Chinese medicine has become an alternative due to its low-toxicity and high-efficacy.

Danggui Buxue Decoction (DBD), a traditional Chinese medicine, has been used for nourishing and enriching the “Blood” for almost 800 years, and its clinical efficacy has been well-documented (Lin et al., 2017). DBD is a simple formula which consists of two botanical drugs: *Radix Angelicae Sinensis* (Danggui, DG) and *Radix Astragali* (Huangqi, HQ) with a weight ratio of 1: 5. A large number of studies have shown that DBD remarkably increases the number of white blood cells, reticulocytes, and bone marrow nucleated cells (Liu et al., 2010; Wang et al., 2020). DBD also significantly affects hematopoietic function by promoting bone tissue regeneration, regulating immune-mediated aplastic anemia, and modulating gut microbiota balance (Yang et al., 2014; Wang et al., 2015; Du et al., 2020). In order to improve the ability to nourish blood, DG and HQ are often used in combination with other botanical drugs in clinical to form modified Danggui Buxue Decoction (MDBD). Traditional Chinese medicine formulas for treating leukopenia, such as Qijiao Shengbai Capsule (Ma et al., 2022) and Qijing Shengbai Granule (Huang, 2019), both contain DG and HQ. Meanwhile, DG and HQ that make up DBD are also the two most frequently used botanical drugs in clinical treatment of aplastic anemia (Dong et al., 2022). Icaritin (ICT) is one of the main effective ingredients of *Herba Epimedii in vivo* which is an important botanical drug that has been used for boosting peripheral blood cells proliferation (Qin et al., 2019). ICT also can improve hematopoietic function of chemotherapy-induced myelosuppression mice by reducing bone marrow depression and improving bone marrow hematopoietic microenvironment (Sun et al., 2018). Our research group has found that ICT may be one of the key pharmacological components of Qijing Shengbai Granule in treating leukopenia in mice (Huang, 2019). In our further research, we found that ICT can exert the best therapeutic effect of increasing white blood cells at 3 mg/kg. ICT is a chemical drug derived from botanical drug, and there are currently no reports of its compatibility with DBD. Meanwhile, it is unclear whether modified Danggui Buxue Decoction (MDBD), consisting of three drugs of DG, HQ and ICT with a weight ratio of 1: 5: 0.003 (DBD: 6 g/kg, ICT: 3 mg/kg), exerts a better efficacy in promoting hematopoiesis.

**FIGURE 1:**
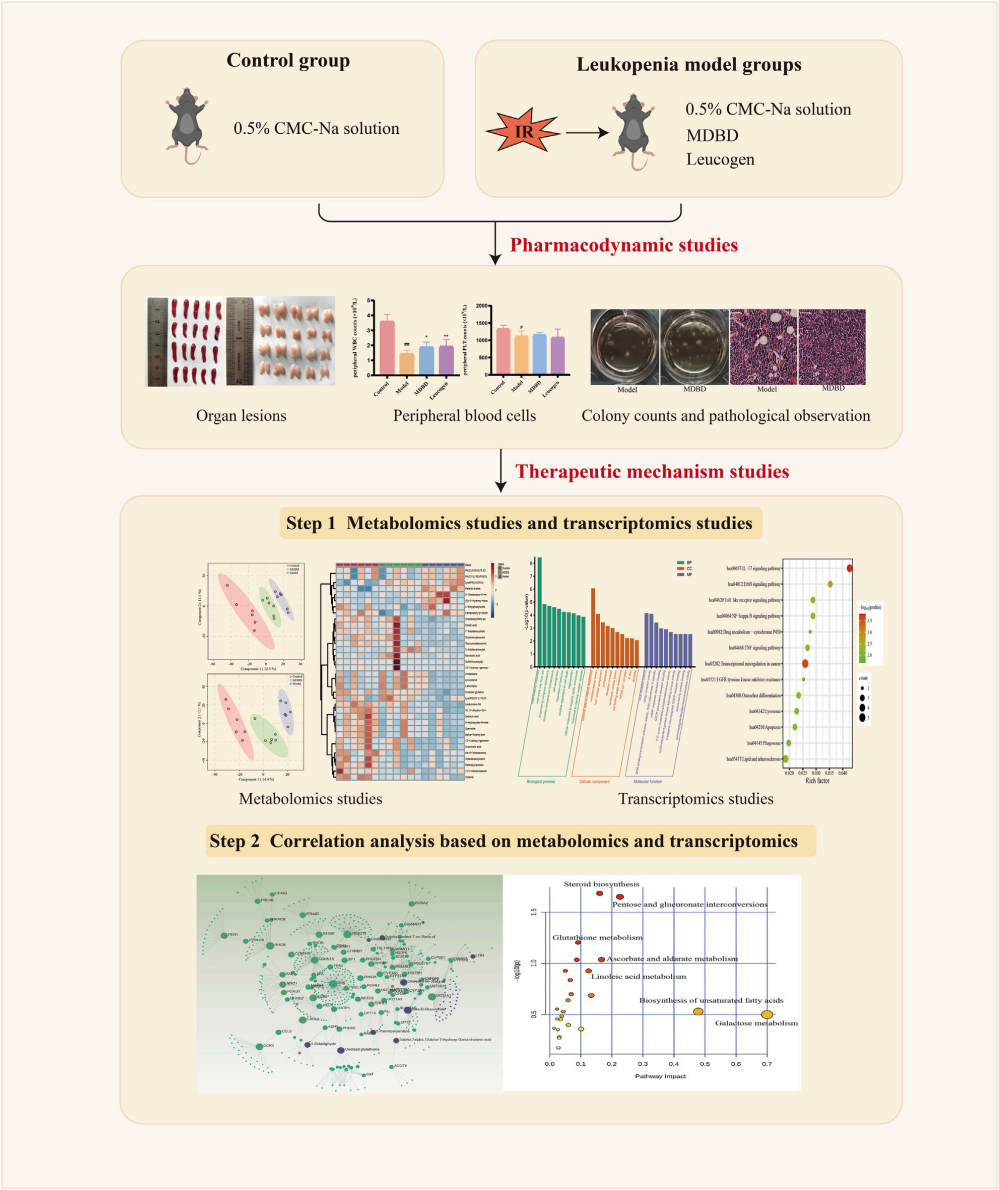
The workflow of protective mechanism of MDBD on radiation-induced leukopenia mice.

In this study, we first studied the effect of MDBD on leukopenia in mice induced by radiation. Next, transcriptomic data from bone marrow and metabonomic data from serum were applied to further study the therapeutic mechanism of MDBD. Finally, we applied correlation analysis based on transcriptomics and metabolomics to explore the mechanism of MDBD in treating leukopenia (Figure 1).

## 2 Materials and methods

### 2.1 Reagents and materials

DG (origin in Gansu, China, batch number 200807), HQ (origin in Gansu, China, batch number 201215) were provided by Shanghai Hongqiao Traditional Chinese Medicine Co., Ltd. (Shanghai, China). And the above two drugs have all reached the characteristic identification standard described in the 2015 edition of the Chinese Pharmacopoeia. ICT (molecular weight = 368.37, Lot: 21398S1, CAS: 118525-40-9, purity ≥98.0%) was offered by Shanghai Sunny Biotech Co., Ltd. (Shanghai, China). Leucogen tablets were purchased from Jiangsu Jibel Pharmaceutical Co., Ltd. (Zhenjiang, China). The ELISA kits for GM-CSF, interleukin-6 (IL-6), thrombopoietin (TPO) were purchased from Abcam (Cambridge, UK). A cobalt radiation source was provided by the Radiation Center of Naval Medical University (Shanghai, China).

### 2.2 Preparation of MDBD

First of all, we prepared DBD with DG and HQ. As described in the literature (Kwan et al., 2019; Shi X. Q. et al., 2020), DBD needs to be extracted with an aqueous solution for optimal efficacy. DG (100 g) and HQ (500 g) were immersed into water (1: 8, w/v) for 2 h, and then decocted in boiling water (1: 8, w/v) for three times, 2 h each time. After filtration, the filtrates were combined and concentrated under reduced pressure (60 °C) to a concentration of 1.2 g mL^-1^. Then, DBD was stored in aliquots at −80 °C for future use. DBD was taken out of the refrigerator and dissolved at 4 °C when using. Then it was diluted with 0.5%CMC-Na solution of equal volume to obtain DBD with a concentration of 0.6 g/mL. Next, ICT powder was dissolved in 0.5% CMC-Na solution to obtain ICT solution with a concentration of 0.3 mg/mL. Finally, we mixed the above DBD solution and ICT solution with equal volume to obtain MDBD, which consisted of three drugs of DG, HQ and ICT with a weight ratio of 1: 5: 0.003.

### 2.3 Establishment of leukopenia model and drug administration

Six-to eight-week-old male C57BL/6 mice (18–22 g) were offered by Shanghai Lingchang Biotechnology Co., Ltd. (Shanghai, China) (SCXK, 2018-0003), and housed in the environmentally controlled breeding room (humidity: 60% ± 5%, temperature: 22°C ± 2 °C). All experiments were approved by the Medical Ethics Committee of Navy Medical University. After a 7-day acclimation period, 40 mice were randomly divided into 4 groups: control group and 3 leukopenia model groups. The three model groups were treated with CMC-Na solution (concentration: 0.5%), MDBD (combine 6 g/kg/day DBD and 3 mg/kg/day ICT) and leucogen (20 mg/kg/day) for 2 days before and for 7 days after radiation, respectively, while the control group was given the same volume of CMC-Na solution by the same gavage method.

Due to the special sensitivity of white blood cells, especially lymphocytes, to radiation, despite individual differences, a dose of 2 Gy can kill 50% of the irradiated population (Pouliliou et al., 2015; Koukourakis and Giatromanolaki, 2021). Meanwhile, some studies have found that in order to observe the response of all blood cells to radiation, especially red blood cells that are not sufficiently sensitive to radiation, the choice of irradiation dose should not be less than 3.5 Gy, and the gender of mice is better for male (Liu et al., 2008). Therefore, in order to obtain a leukopenia model, leukopenia model mice received a 350 cGy total body radiation at the rate of 32.41 cGy/min. Mice in the control group underwent a sham radiation procedure.

### 2.4 Pharmacodynamics of MDBD

#### 2.4.1 Measurement of peripheral blood cells and organ indexes

On the 14th day after administration, 100 μL of whole blood was collected from the eyeballs with anticoagulant EP tubes after the mice were anesthetized with 4% chloral hydrate (0.05 mL/10 g), and blood routine examination was performed with the hematology analyzer (Mindray, BC-5000Vet, China). The remaining eyeballs blood was collected continuously with EP tubes without anticoagulant for subsequent experiments. Thymus and spleen are important immune organs in mammals, and both of them have lesions in anemic mice (Shi X. Q. et al., 2020). The mice were sacrificed after blood collection, and the spleen and thymus were collected and weighed to calculate the organ index.

#### 2.4.2 Determination of bone marrow cells

Bone marrow cells were collected from femurs for bone marrow nuclear cells (BMNCs) count and cell viability assays. After the mice were sacrificed, they were immersed in 75% alcohol for 3 min immediately. Then, the unilateral femur was removed under sterile conditions. The muscle and connective tissue on the femur were removed as many as possible. Next, carefully cut open both ends of the femur with sterile scissors. Bone marrow cells were flushed from the bone marrow cavity into a sterile EP tube with 1 mL DMEM medium and rinsed 4 to 6 times repeatedly. Afterwards, bone marrow cells were pipetted into a single-cell suspension, resuspend and counted in automated cell counter.

Cell viability assays were performed with Cell Counting Kit-8 reagent (Adamas, Switzerland). BMNCs (100 µL/well) were added to 96-well culture plates at a density of 1 × 10^7^/mL and then incubated with 10 µL CCK-8 reagent at 37 °C for 4 h. Lastly, the absorbance was measured at 450 nm.

#### 2.4.3 Colony-forming units assay

After the cell viability was determined, the remaining cell suspension was filtered through a 70 μm cell strainer. We slowly added the filtered cell suspension to the upper layer of Ficoll lymph separation solution of equal volume, and centrifugated at 2000 r/min for 20 min. Additionally, we carefully suck out the middle milky white cloud layer (bone marrow mononuclear cells), and added 5 times of the volume of DMEM medium to wash twice. Bone marrow cells were diluted with M3434 methylcellulose medium (StemCell, Canada) to a density of 2 × 10^4^/mL. Then the cells were evenly spread in the middle four wells of the 12-well culture plate (with 2 mL PBS added to the surrounding eight wells) with 1 mL per well, and cultured at 37 °C, 5% CO_2_ and saturated humidity for 10 days. Finally, we counted the number of colonies under the microscope with different magnification, and the cell cluster containing more than 50 cells was counted as one colony.

#### 2.4.4 ELISA analysis

The blood samples in the EP tube without anticoagulant were placed at room temperature and clotted for 2 h, then centrifuged at 2000 r/min for 15 min. The serum was collected and divided equally, and stored in the −80 °C refrigerator for future use. GM-CSF, TPO and IL-6 in serum were quantified by ELISA kit according to the manufacturer’s instructions, and optical density of each sample was measured at 450 nm. Finally, the cytokine level was quantified by standard curve and expressed in pg/mL.

#### 2.4.5 Effects of MDBD on bone marrow histology

Four unilateral femurs of each group of mice were stained with hematoxylin-eosin (H&E) to make pathological sections of bone marrow. Morphological and pathological observation and analysis were carried out under the microscope. Five random visual fields were selected for each femur slice, and the percentage of hematopoietic area in bone marrow sections was measured with image analysis software (Image J) to judge the structural impact of MDBD on bone marrow tissue.

### 2.5 Untargeted metabolomics analysis

#### 2.5.1 Serum pretreatment and UPLC-Q/TOF-MS analysis

Six serum samples from mice in control, model and MDBD group were randomly selected and thawed at 4 °C before metabolomics analysis. Each serum sample (100 μL) was mixed with four times the amount of methanol and swirled for 5 min, then centrifuged at 12000 rpm for 10 min for protein precipitation. Next, the supernatant was transferred to auto-sampler vials for ultra-performance liquid chromatography coupled with quadrupole time-of-flight mass spectrometry (UPLC-Q/TOF-MS) analysis. At the same time, we took an equal amount of 10 μL supernatant from each sample and mixed it into QC sample, which were injected once every six samples to monitor the repeatability and stability of instrument.

Chromatographic separation was executed by using an ACQUITY UPLC system (Waters Corp., Milford, United States) with a ACQUITY UPLC HSS T3 (2.1 × 150 mm, 1.8 µm particles, Waters Corp., Milford, United States) chromatographic column with a temperature maintenance of 40 °C. The serum sample injection volume was set to 3 µL and the temperature of autosampler was fixed at 8 °C. The gradient mobile phase was a mixture of 0.1% formic acid in water (phase A) and acetonitrile (phase B), which was pumped at a flowing rate of 0.4 mL/min. The optimal elution procedure of phase B was set as follows: 0–0.5 min, 5%; 0.5–5 min, 5%–70%; 5–11 min, 70%–75%; 11–14 min, 75%–95%; 14–14.5 min, 95%–5%; 14.5–17 min, 5%. Mass spectrometry data detection and acquisition was performed by SYNAPT G2-Si time-of-flight mass spectrometry (Waters Corp., Milford, United States) coupled with an electrospray ionization source (ESI). Mass spectra obtained in positive and negative ion mode respectively. The temperature of ESI source was set at 120 °C, and the capillary voltage was 2.0 kV. The desolvation gas (nitrogen) temperature was 400 °C with a flow rate of 800 L/h. The flow rate of cone gas was 50 L/h. Mass data were gathered between 50 and 1,000 m*/z* with the 0.2 s scanning time. Leucine enkephalin (LE) was used as the external reference (LockSpray™) to ensure the precision and accuracy of mass information. The flow rate of LE was 5 μL/min with a concentration of 1 μg/mL. The MS collision energy was set from 10 to 45 V.

#### 2.5.2 Data analysis

Raw serum data were performed using Progenesis QI (V2.0, Nonlinear Dynamics Ltd., Newcastle, UK). Next, the pre-processed data were imported into SIMCA 14.1 (Umetric, Umeå, Sweden) and MetaboAnalyst 5.0 for multivariate statistical analysis. At the same time, differential metabolite ions with VIP >1.5 in the OPLS-DA model, *p*-value <0.05 in *t*-test and FC > 1.5 were selected as candidates. After that, the candidate metabolites were screened and identified based on by Human Metabolome database (HMDB) and the Kyoto Encyclopedia of Genes and Genomes (KEGG). Finally, MetaboAnalyst 5.0 was used to analyze the pathway of the screened differential metabolites.

### 2.6 Transcriptomic analysis

#### 2.6.1 RNA extraction, library construction, and sequencing

Femoral bone marrow cells from 18 mice in control group, model group and MDBD group (n = 6) were selected for transcriptomic analysis. The total RNAs were isolated from bone marrow cells and purified using Trizol (Beyotime, Shanghai, China) reagent following the manufacturer’s instruction. Nanodrop 2000 (NanoDrop, Wilmington, DE, United States) was applied to detect the concentration and purity of the extracted RNA. Meanwhile, RNA integrity was detected by agarose gel electrophoresis, and RIN value was determined by Agilent 2,100 system (Agilent Technologies, CA, United States). The total amount of RNA was required to be ≥1 μg and the concentration ≥35 ng for a single database establishment. Sequencing libraries were constructed using Illumina Truseq™ RNA library Prep kit (Illumina, Nebraska, United States) according to manufacturer’s protocol.

#### 2.6.2 Data analysis and enrichment analysis for gene expression

The data were uploaded to the online platform of Majorbio Cloud Platform (www.majorbio.com). Differential expression analysis was conducted by DESeq2 software. The significantly different expression genes (DEGs) between groups were determined according to the following criteria: *p*-value ≤0.05 and |log2FoldChange| ≥ 0.585.

In addition, functional-enrichment analysis including GO (Gene Ontology) and KEGG were performed to identify which DEGs were significantly enriched in GO terms and metabolic pathways at *p*-value ≤0.05 compared with the whole-transcriptomic background. GO functional enrichment and KEGG pathway analysis were carried out by Metascape (https://metascape.org). Finally, OmicsNet 2.0 (https://www.omicsnet.ca) was used for correlation analysis of metabolomics and transcriptomics.

### 2.7 RNA isolation and quantitative real-time PCR

Total RNA was extracted from bone marrow cells of control group, model group and MDBD group with RNAiso Plus (Takara, Japan). Then PrimeScript Master Mix kit (Takara, Japan) was used to generate cDNA. Quantitative real-time PCR (qRT-PCR) was performed using PowerUp™ SYBR™ Green Master Mix (Thermo Fisher, United States) and QuantStudio3 (Applied Biosystems) according to protocol. The primer sequences (Supplementary Table S1) were designed by BioTNT Co., Ltd. (Shanghai, China). The mRNA expression levels of target genes were normalized with the reference gene *β*-actin, and then the results were analyzed using 2^−ΔΔCT^ method.

### 2.8 Statistical analysis

The data was depicted as the means ± SD (standard deviation). Statistical analyses of multiple groups were conducted via one-way ANOVA (one-way analysis of variance) by using the GraphPad Prism 8 software. When *p*-value was less than 0.05, it was considered statistically significant. In PPI analysis, the species were limited to *Homo sapiens* with a minimum required interaction score of 0.400 (medium confidence), and disconnected nodes in the network were hidden.

## 3 Results

### 3.1 MDBD increased peripheral WBC counts and spleen index

**FIGURE 2:**
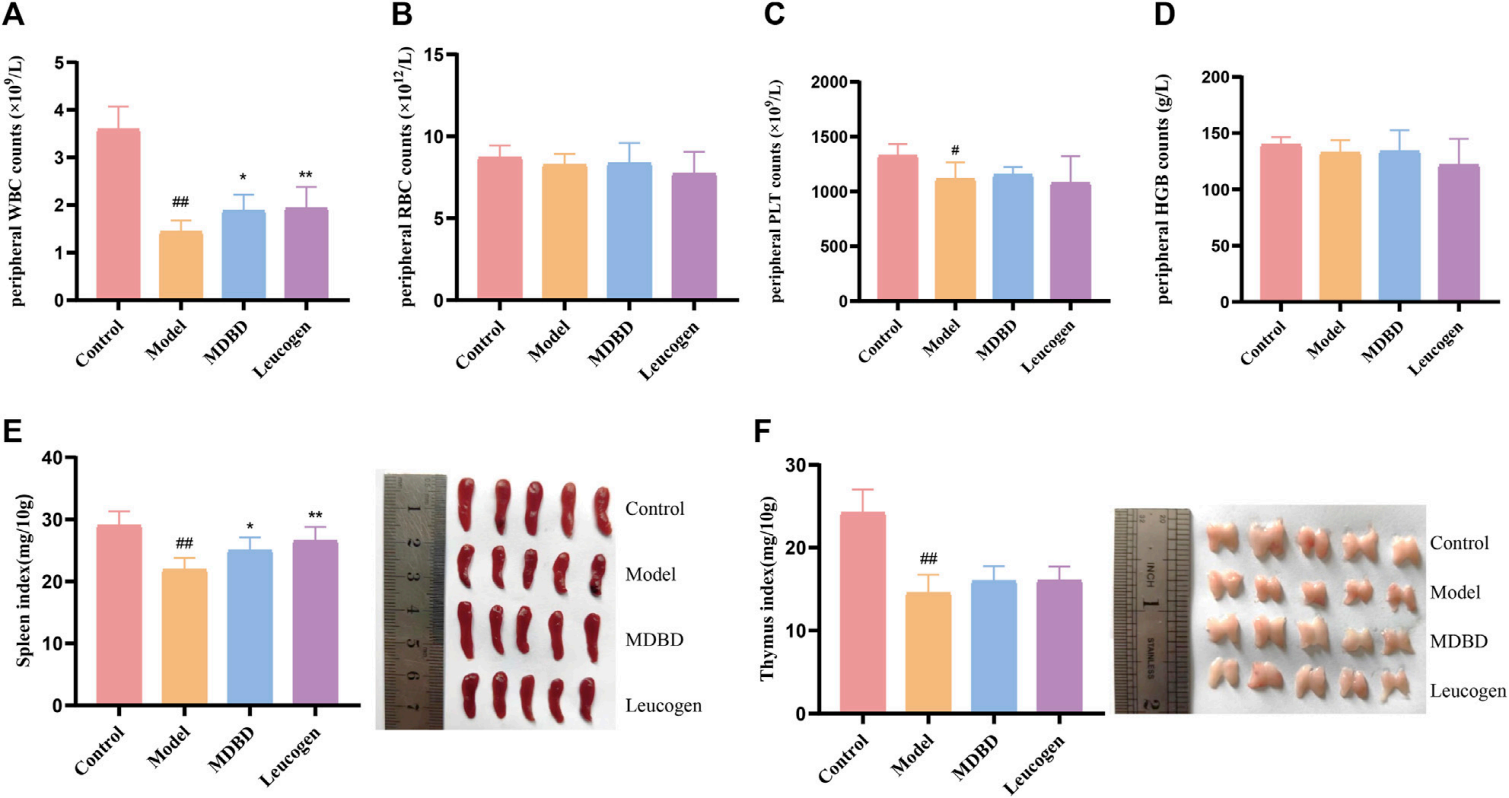
Effect of MDBD on peripheral blood cells and organ indexes in leukopenia mice induced by radiation. **(A)** WBC. **(B)** RBC. **(C)** PLT. **(D)** HGB. **(E)** Spleen index. **(F)** Thymus index. After treatment with MDBD, WBC **(A)** and spleen index **(E)** increased compared with model group. The data are expressed as means ± SD. #*p* < 0.05 and ##*p* < 0.01 vs control group; *p < 0.05 and **p < 0.01 vs. model group.

It is shown in Figures 2A–D that compared with the control group, peripheral WBC (*p* < 0.01) and PLT (*p* < 0.05) were significantly decreased in the model group, which demonstrated that a model of radiation-induced leukopenia was successfully established. After MDBD treatment, the WBC content increased obviously (*p* < 0.05), and PLT also tended to increase (no statistical significance). However, no significant changes were observed in peripheral RBC and HGB under given radiation dose. The differential cell count of WBC showed that radiation could significantly reduce the counts of neutrophils, lymphocytes, monocytes, and eosinophils, but had no significant effect on the counts of basophils and the proportion of each type of cells (Supplementary Figure S1). Furthermore, the administration of MDBD did not significantly change the proportion and quantity of leukocyte cells of each type caused by radiation (Supplementary Figure S1). Compared with the control group, the spleen index and thymus index were significantly decreased (*p* < 0.01) in the model group, which means spleen damage and thymus atrophy. Meanwhile, MDBD could reverse the spleen index to the control level (*p* < 0.05) but had no visible effect on the thymus index (Figures 2E,F).

### 3.2 MDBD increased bone marrow nucleated cells and enhanced cell viability

**FIGURE 3:**
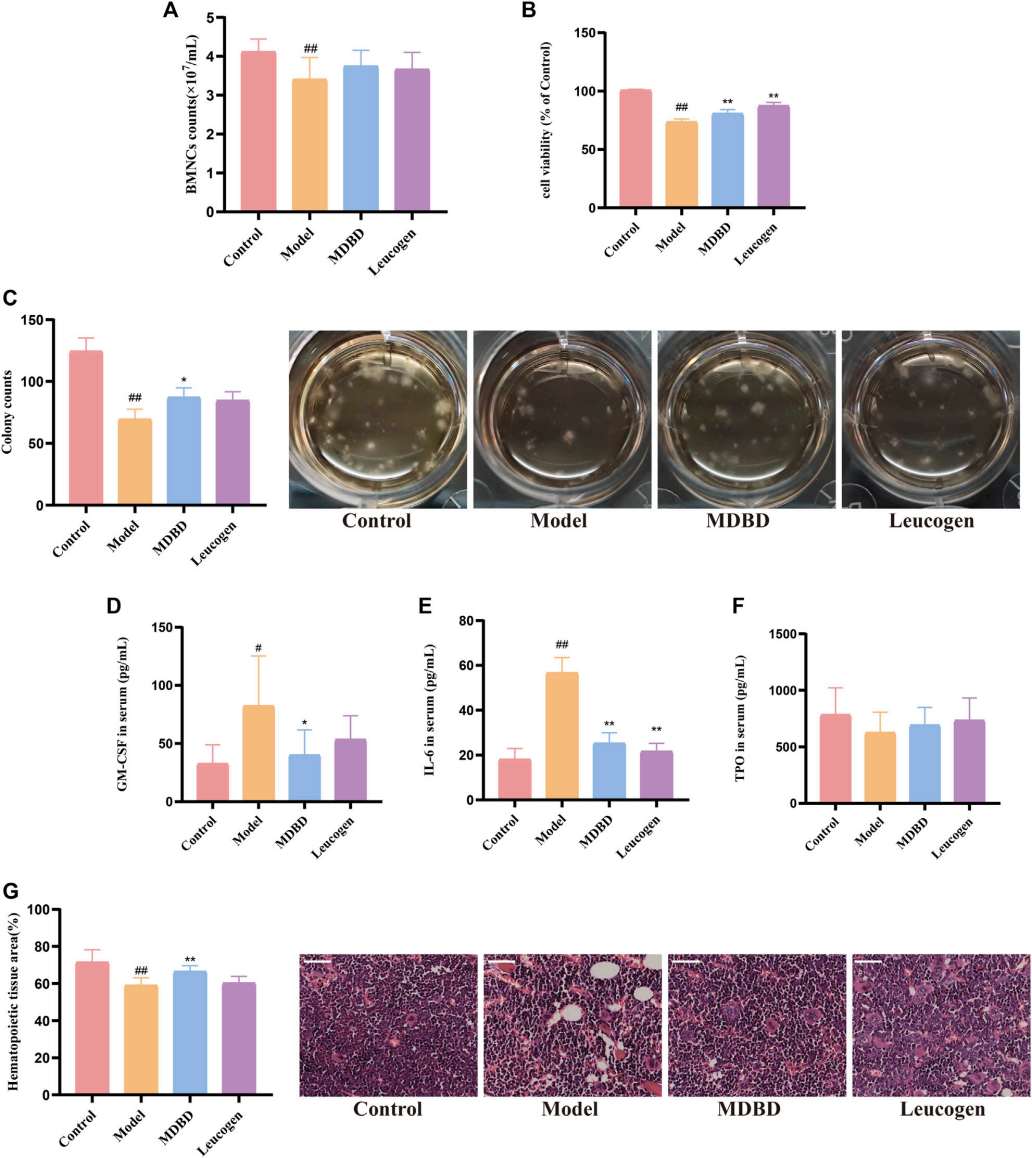
The effect of MDBD on the cell number and viability of BMNCs, colony-forming assay of bone marrow mononuclear cells, cytokines concentration and hematopoietic area. **(A)** BMNCs counts. **(B)** Cell viability. **(C)** Colony forming of bone marrow mononuclear cells. **(D)** GM-CSF. **(E)** IL-6 **(F)** TPO. **(G)** Hematopoietic area. The scale in the figures represents 50 μm. The data are expressed as means ± SD. #*p* < 0.05 and ##*p* < 0.01 vs control group; *p < 0.05 and **p < 0.01 vs model group.

Peripheral blood cells are derived from hematopoietic stem cells in bone marrow, and the number of BMNCs indirectly reflects hematopoietic function. Compared with the control group, the count and the cell viability of BMNCs were significantly reduced in the model group (*p* < 0.01), reflecting the myelosuppressive effect of radiation. After administration of MDBD, the cell viability enhanced significantly (*p* < 0.01), while the increase of BMSCs count was not significant (*p* > 0.05) (Figures 3A,B).

### 3.3 MDBD promoted colony formation of bone marrow mononuclear cells

To detect the effect of MDBD on hematopoietic colony formation unit (CFU) *in vivo*, the number of erythroid (CFU-E), myeloid (CFU-GM), megakaryocytic (CFU-MK), mixed (CFU-GEMM) lineages and bone marrow stromal cells (CFU-F) clusters were counted together under the microscope on the 10th day of culture. CFU of bone marrow mononuclear cells was obviously decreased by radiation (*p* < 0.01). After treatment with MDBD, the CFU count increased significantly (*p* < 0.05) (Figure 3C).

### 3.4 MDBD regulated hematopoiesis-related cytokines levels

Hematopoiesis-related cytokines such as GM-CSF, TPO and IL-6 are important factors regulating hematopoietic function (Liu et al., 2021). The levels of GM-CSF, TPO and IL-6 in serum were determined by ELISA kits. Compared with control group, the levels of GM-CSF and IL-6 in serum were significantly increased respectively after radiation treatment. MDBD reversed the dramatically change of GM-CSF and IL-6. In addition, no significant changes in serum TPO levels were observed (Figures 3D–F).

### 3.5 MDBD increases the area of bone marrow hematopoietic tissue

In the bone marrow of control group, the distribution of erythroid and granulocyte cells was relatively tight and uniform with intact structure of hematopoietic scaffold, and the proportion of hematopoietic area was more than 70%. Compared with the control group, the hematopoiesis area of the model group decreased obviously concomitant with an increase of vacuoles and adipocytes cells (*p* < 0.01), which indicated that radiation significantly inhibited hematopoiesis. Furthermore, the adipocytes and vacuoles in the MDBD group were reduced compared with the model group accompanied by the structural integrity of the hematopoietic scaffold was improved, and the hematopoietic area was significantly increased (*p* < 0.01) (Figure 3G).

### 3.6 Metabolomics studies

**FIGURE 4:**
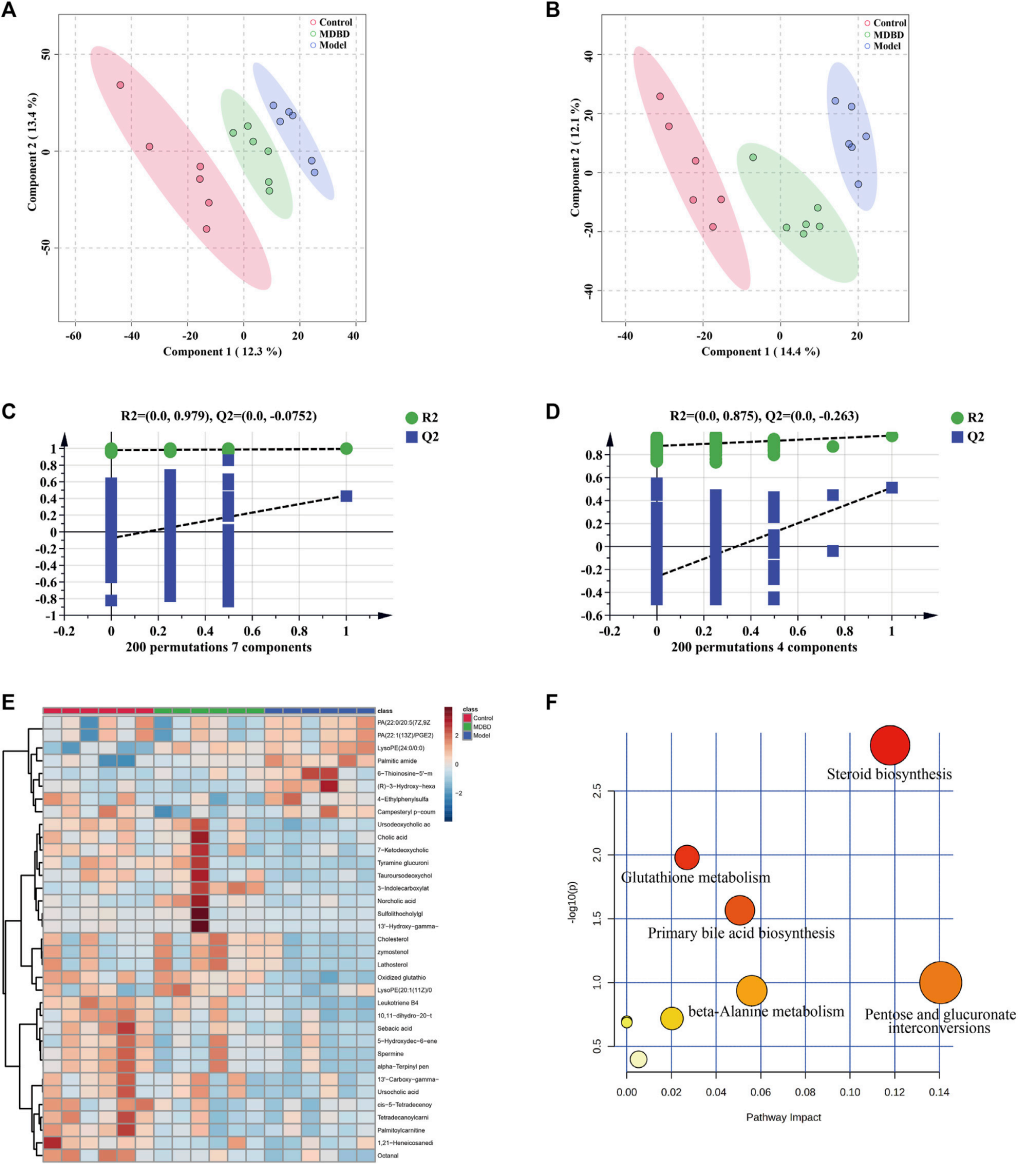
MDBD modulated the metabolites in serum of radiation-induced leukopenia mice. **(A)** PLS-DA score plots in ESI + mode. **(B)** PLS-DA score plots in ESI- mode. **(C)** Permutation test of PLS-DA in ESI + mode. **(D)** Permutation test of PLS-DA in ESI- mode. **(E)** Clustering heatmap of potential differential metabolites in serum. **(F)** The key metabolic pathways of differential metabolites.

In order to distinguish the metabolic profiles among control, model and MDBD group, PCA and PLS-DA in ESI^+^ mode (positive ion mode) and ESI^−^ mode (negative ion mode) were performed for cluster analysis. The serum samples between the control, model, and MDBD groups tended to be separated in PCA (Supplementary Figure S2). Meanwhile, the significant separation of different groups in PLS-DA confirmed the reliability and reproducibility of the test method (Figures 4A,B). Furthermore, the permutations plot of PLS-DA showed that the model was non-overfitting and reliable (Figures 4C,D). Metabolites with VIP >1.5, *p* < 0.05 and FC > 1.5 were selected and considered as potential metabolites by matching with substance molecules in HMDB. 35 differential metabolites (Supplementary Table S2) were identified among the control, model and MDBD groups. After administration of MDBD, compared with the model group, all 35 metabolites were inversely regulated by MDBD, and 14 metabolites of them were significantly regulated by MDBD (*p* < 0.05). To further capture the differences of metabolites among three groups, a heatmap was drawn (Figure 4E). In addition, in order to identify the key metabolic pathways, MetaboAnalyst 5.0 was applied for metabolic pathway enrichment analysis. The main metabolic pathways were enriched as follows: steroid biosynthesis, glutathione metabolism, primary bile acid biosynthesis, pentose and glucuronate interconversions, beta-alanine metabolism, arachidonic acid metabolism, arginine and proline metabolism, fatty acid degradation, drug metabolism-other enzymes and steroid hormone biosynthesis (Figure 4F; Table 1).

**TABLE 1:** Pathways enrichment analysis of differential metabolites in serum of MDBD-treated mice.

NO.	Term	Match status	*p*-value	-Log(*p*)	FDR
1	Steroid biosynthesis	3/42	0.0019464	2.7108	0.1635
2	Glutathione metabolism	2/28	0.012953	1.8876	0.54401
3	Primary bile acid biosynthesis	2/46	0.033327	1.4772	0.93315
4	Pentose and glucuronate interconversions	1/18	0.11055	0.95644	1
5	beta-Alanine metabolism	1/21	0.12786	0.89325	1
6	Arachidonic acid metabolism	1/36	0.20998	0.67783	1
7	Arginine and proline metabolism	1/38	0.22038	0.65683	1
8	Fatty acid degradation	1/39	0.22554	0.64678	1
9	Drug metabolism - other enzymes	1/39	0.22554	0.64678	1
10	Steroid hormone biosynthesis	1/85	0.43203	0.36449	1

### 3.7 Transcriptomics studies

**FIGURE 5:**
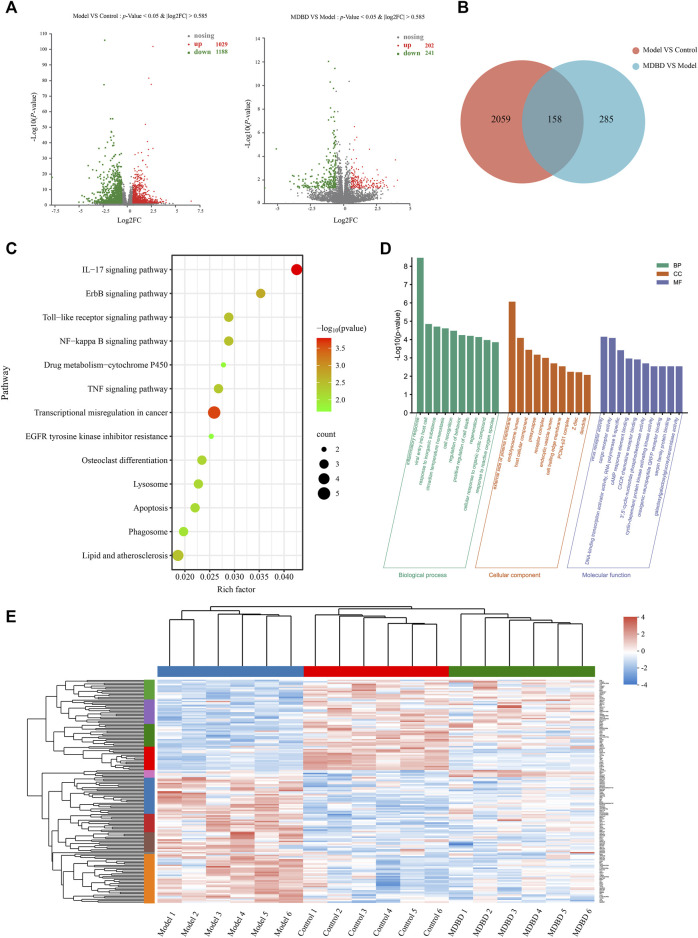
Transcriptomics analysis of MDBD on radiation-induced leukopenia mice. **(A)** Relative volcano plot in different groups. **(B)** Veen diagram of differently expressed genes among control, model and MDBD groups. **(C)** KEGG enrichment analysis of differently expressed genes. **(D)** GO enrichment analysis of differently expressed genes. **(E)** Clustering heatmap of DEGs.

A total of 2,217 DEGs between model and control group were identified, including 1,029 upregulated and 1,188 downregulated genes (Figure 5A). Meanwhile, 443 DEGs were identified between MDBD treatment and model group, which included 202 upregulated and 241 downregulated genes (Figure 5A). In order to find out DEGs regulated by MDBD, we crossed all the above differential genes and got 158 specific DEGs (Supplementary Table S3, Figure 5B), which were inversely expressed by 148 after MDBD treatment. To further visualize the differences among the DEGs among three groups, a heatmap was drawn (Figure 5E). In order to identify the potential pathway of MDBD, the above 158 specific DEGs were performed for KEGG pathway enrichment analysis. These pathways were mainly enriched in IL-17 signaling pathway, ErbB signaling pathway and Toll-like receptor signaling pathway (Figure 5C). To further understand the cellular processes and function of the DEGs, GO terms enrichment analysis was performed. The GO terms mainly mapped to inflammatory response of BP (biological processes), external side of serum membrane of CC (cell components), and virus receptor activity of MF (molecular functions) (Figure 5D). The results of enrichment analysis indicated that MDBD may influence these biological processes and pathways to increase peripheral WBC in radiation-induced leukopenia mice.

### 3.8 Integrated analysis of MDBD-Treated radiation-induced leukopenia mice from metabolomics and transcriptomics data

**FIGURE 6:**
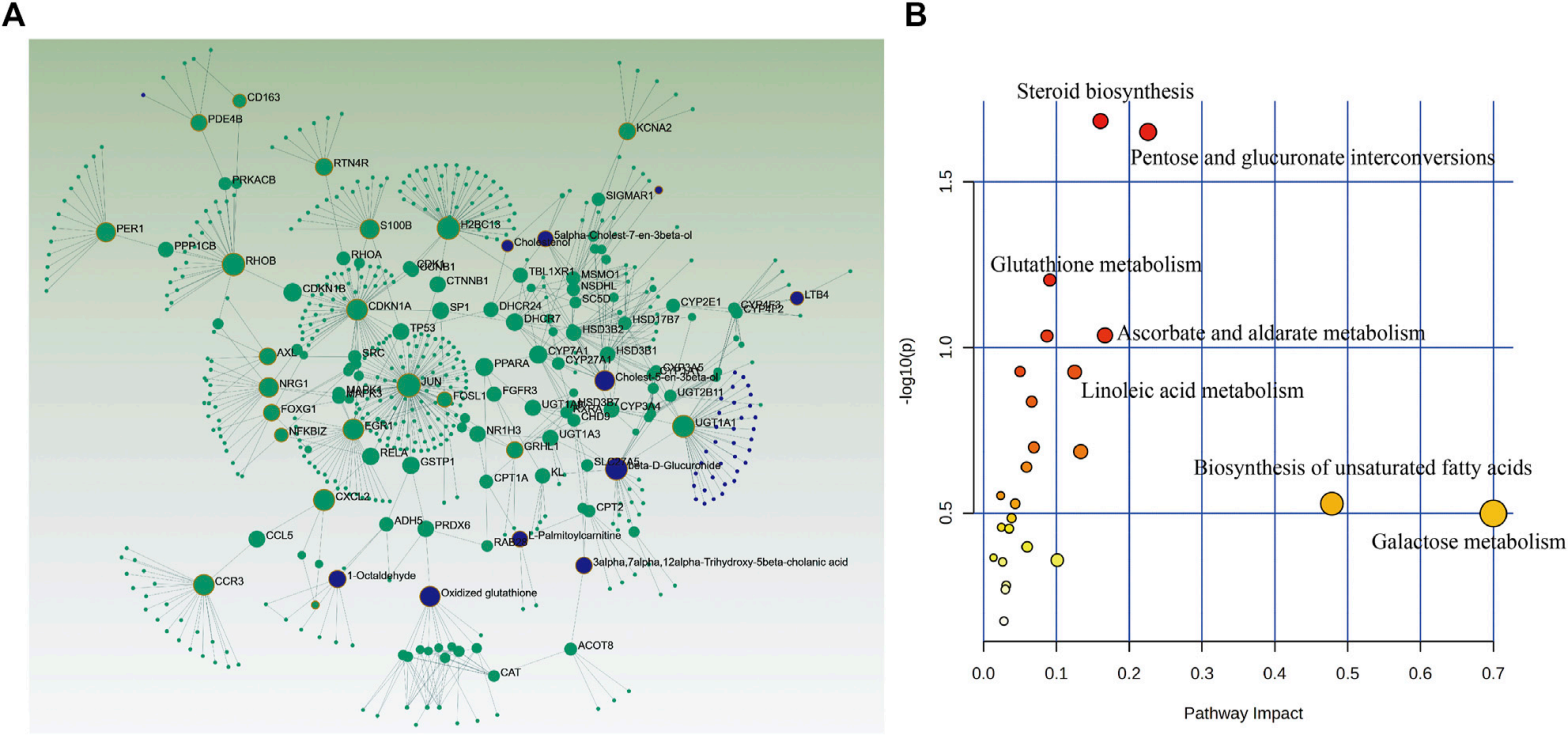
Integrated analysis of the differential metabolites and differently expressed genes using OmicsNet 2.0 and MetaboAnalyst 5.0. Metabolites (blue) and genes (green) are presented as nodes and relationships are presented as edges. The input genes and metabolites have a yellow outer circle when having a critical role. **(A)** Integrated analysis network. **(B)** Joint-pathway analysis.

In order to further explore the relationship between DEGs and candidate metabolites, 158 DEGs and 35 differential metabolites were uploaded to OmicsNet 2.0 and MetaboAnalyst 5.0 to obtain the potential relationship between DEGs and candidate metabolites regulated by MDBD. The results of metabolites-genes association network analysis finally focused on the steroid biosynthesis, pentose and glucuronate interconversions, glutathione metabolism (Figures 6A,B; Table 2), among which the key genes were Jun, Cxcl2 and Egr1, as these genes had relatively highest scores. At the same time, in transcriptome analysis, through PPI analysis of 158 DEGs, we can also focus on genes Cxcl2, Egr1 and Jun as key genes (Supplementary Figure S4). The integrated analysis suggested that these pathways and genes may be the key biological process and genes for MDBD to increase WBC in radiation-induced leukopenia mice.

**TABLE 2:** Integrated analysis of 158 DEGs and 35 differential metabolites.

NO.	Term	Total	Expected	Hits	-Log(*p*)
1	Steroid biosynthesis	82	0.60492	3	1.6833
2	Pentose and glucuronate interconversions	32	0.23607	2	1.6503
3	Glutathione metabolism	56	0.41311	2	1.2035
4	Ascorbate and aldarate metabolism	13	0.095902	1	1.0363
5	Drug metabolism-other enzymes	70	0.51639	2	1.0343
6	Arachidonic acid metabolism	81	0.59754	2	0.92718
7	Linoleic acid metabolism	17	0.12541	1	0.92576
8	Primary bile acid biosynthesis	92	0.67869	2	0.83637
9	Mannose type O-glycan biosynthesis	30	0.22131	1	0.69841
10	Glycosphingolipid biosynthesis-globo and isoglobo series	31	0.22869	1	0.68564
11	Glycerolipid metabolism	35	0.2582	1	0.63883
12	beta-Alanine metabolism	44	0.32459	1	0.55261
13	Retinol metabolism	47	0.34672	1	0.52833
14	Biosynthesis of unsaturated fatty acids	47	0.34672	1	0.52833
15	Galactose metabolism	51	0.37623	1	0.49866

### 3.9 MDBD regulate the gene expression of Cxcl2, Egr1 and Jun

**FIGURE 7:**
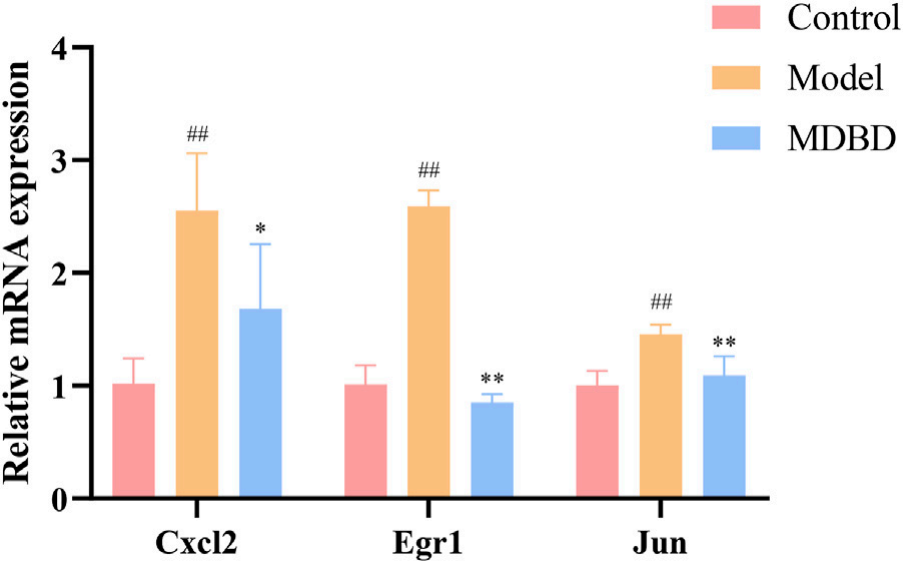
The effects of MDBD on the expression of the key genes (Cxcl2, Egr1 and Jun) in bone marrow cells from radiation-reduced leukopenia mice. The data are expressed as means ± SD. #*p* < 0.05 and ##*p* < 0.01 vs. control group; *p < 0.05 and **p < 0.01 vs. model group.

In order to further study the effects of MDBD on the regulation of key metabolic pathways and the accuracy of the multi-omics integrated analysis, we detected the expression of Cxcl2, Egr1 and Jun, which were key genes in the integrated analysis. The bone marrow cells of radiation-induced leukopenia mice were analyzed by qRT-PCR. Compared with the control group, the expression of Cxcl2, Egr1 and Jun in the model group increased significantly. QJSB significantly regulated the mRNA level of these genes (Figure 7). In addition, the results of qRT-PCR analysis were consistent with those of transcriptomics results, which show the reliability of our conclusions.

## 4 Discussion

Since the first clinical application of radiotherapy in 1896, radiotherapy has undergone tremendous development (Allen et al., 2017). Currently, radiotherapy is one of the main treatment schedules for approximately 50% of cancer patients, whether used alone or in combination with chemotherapy and surgery to treat a wide range of malignant tumors (Delaney et al., 2005; Citrin, 2017). At the same time, radiation treatments may have chronic or acute side effects, which limit the sustainability of treatment and affect the quality of life, and leukopenia is one of the most common and costly complications (Jairam et al., 2019). As a hematopoietic drug, the substantial efficacy of DBD has been confirmed by a large number of experiments and clinical data (Yang et al., 2009; Zheng et al., 2010). In this study, we successfully demonstrated that the combination of DBD and ICT also could alleviate radiation-induced leukopenia. In our further study (Supplementary Figure S3), it was found that the effect of increasing WBC with MDBD was better than using DBD or ICT alone, and the effect of medium dose MDBD (6 g/kg DBD +3 mg/kg ICT) was better than that of low dose MDBD (3 g/kg DBD +1.5 mg/kg ICT) and high dose MDBD (12 g/kg DBD +6 mg/kg ICT), which suggests that there may be a synergistic effect between DBD and ICT when used together, thereby improving the efficacy.

In addition, hematopoietic-related cytokines, such as GM-CSF, IL-6 and TPO, had also been detected in the pharmacodynamic study of MDBD due to their important regulatory effects on the hematopoietic system. GM-CSF was originally discovered to be a protein capable of generating granulocyte and macrophage colonies from myeloid precursor cells *in vitro* (Becher et al., 2016). Meanwhile, it was reported that GM-CSF, IL-6 and TPO were regulated by DBD in myelosuppression mice (Liu et al., 2021). However, the over-expression of GM-CSF will over-mobilize HSCs in the temporary non-proliferation phase, which will make them enter the cell proliferation cycle prematurely, resulting in the excessive production and activation of granulocytes and macrophages, ultimately reducing the body’s hematopoietic function, and even inducing a variety of hematological diseases (Dhagat et al., 2018). At the same time, studies have shown that excessive secretion of IL-6 will also destroy the homeostasis of HSCs (hematopoietic stem cells), leading to damage to the body’s hematopoietic function (O'Hagan-Wong et al., 2016). In our pharmacodynamic study, MDBD reversed the excessive depletion of certain cytokines caused by radiation, which may contribute to the recovery of leukopenia. The mammalian spleen is considered as a secondary peripheral lymphoid organ and a red blood cells bank, which plays an important role in immunity and hematopoiesis (Brendolan et al., 2007). Our study showed that the spleen index significantly improved after administration of MDBD, indicating that MDBD may participate in the regulation of hematopoietic and immune functions to alleviate radiation-induced leukopenia.

HSCs are maintained in the specialized niches of bone marrow throughout life, and long-term maintenance of HSCs is achieved by balancing self-renewal or differentiation with signals that promoted cell division or quiescence (Cordeiro Gomes et al., 2016). Interestingly, MDBD increased cell viability and promoted colony formation of bone marrow mononuclear cells but did not change the number of BMNCs. This indicated that MDBD indirectly increased the hematopoietic capacity of bone marrow cells without changing the quantity. The pharmacodynamic results fully showed that the MDBD designed by our research group could effectively treat radiation-induced leukopenia.

To further explore the potential mechanism of MDBD in the treatment of leukopenia, we adopted metabolomics and transcriptomics techniques. Metabolomics demonstrated that the regulated metabolites were mainly involved in steroid biosynthesis, glutathione metabolism and primary bile acid biosynthesis. As an important derivative of cholesterol, steroids play an active role in regulating the water-salt balance, stress response, metabolism, and in maintaining sexual differentiation (Schiffer et al., 2019). When Xie et al. (Xie et al., 2016) studied the perimenopausal syndrome caused by estrogen deficiency, he found that the modified Danggui Buxue Decoction composed of DG, HQ and *Herba Epimedii* could significantly alleviate the disorder of steroid hormone metabolism in rat serum. Studies suggested that DBD can play the role of estrogen, and the effect of alleviating menopausal syndrome is mainly caused by the calycosin in HQ (Gong et al., 2016). The redox homeostasis of mitochondria is regulated by the rapidly reactive antioxidant system, and glutathione is one of the key substances to maintain the redox homeostasis of mitochondria and repair mitochondrial damage (Mari et al., 2009). The significant regulation of MDBD on glutathione pathway suggests that MDBD may reduce radiation-induced bone marrow injury by regulating the repair of mitochondrial damage, thereby increasing the number of white blood cells in peripheral blood. Bile acid is synthesized by cholesterol in the liver through multi-step enzymatic reaction, which can stimulate the peristalsis of duodenum and colon, and plays an important role in fat metabolism and glucose metabolism (Jia et al., 2018). Study shown that DBD can regulate the homeostasis of intestinal microflora by regulating the biosynthesis of primary bile acids and improve the metabolic abnormalities in mice. It was shown that DBD can regulate the homeostasis of intestinal microflora by regulating the biosynthesis of primary bile acids and improve the metabolic abnormalities in mice (Du et al., 2020). This results also indicate that MDBD may also be involved in regulating intestinal microflora.

Transcriptomics showed that the regulated differential genes mainly participated in IL-17 signaling pathway and ErbB signaling pathway. IL-17 is mainly an inflammatory factor secreted by CD4 T-cell (Th17), which can induce fibroblasts, keratinocytes, endothelial cells, epithelial cells, *etc.*, to synthesize and secrete inflammatory factors such as G-CSF, IL-6, IL-8, MCP-1, PGE2, and is closely related to asthma, rheumatoid arthritis, lupus and other inflammatory diseases (Park et al., 2005). The main function of IL-17 is to induce G-CSF and IL-8 (Kolls and Linden, 2004), as well as chemokine Cxcl1 and Cxcl2 (Onishi and Gaffen, 2010), and participate in the recruitment of neutrophils during tissue inflammation, and finally attract neutrophils and other myeloid cells to the injured tissue to cause inflammatory reaction. Mice with IL-17 receptor deficiency will weaken the host’s defense against microbial infection due to the significant reduction of G-CSF in the lung (Ye et al., 2001). Some studies also found that when IL-17 is deficient, mice will show increased resistance to arthritis (Nakae et al., 2003). The pro-inflammatory ability of IL-17 is the key to its protection, but when the signal pathway of IL-17 is disordered, it may lead to a series of diseases, showing both pathogenic and protective effects on the body (Amatya et al., 2017). The enrichment of IL-17 pathway in transcriptome results indicates that MDBD may participate in inflammatory reaction.

Additionally, bioinformatics analysis indicated that steroid biosynthesis, pentose and glucuronate interconversions, glutathione metabolism may be the key pathways where MDBD exerted its efficacy against leukopenia. The steroid biosynthesis pathway, as the most critical pathway in bioinformatics analysis, has also been proven to be crucial in metabolomics analysis. After irradiation treatment, the content of the three important intermediate products involved in steroid biosynthesis (Cholest-5-en-3beta-ol, Lathosterol, and Cholesterol) decreased. However, MDBD administration can significantly increase the content of these three intermediate products. This result suggests that the steroid biosynthesis pathway may be one of the key pathways in the treatment of leukopenia with MDBD.

In addition, beta-D-Glucuronide, Cholest-5-en-3beta-ol and Oxidized glutathione were the main metabolites and Jun, Cxcl2 and Egr1 were the main genes. As important participants of energy metabolism *in vivo*, beta-D-Glucuronide and Cholest-5-en-3beta-ol metabolic disorder were regulated by MDBD. Oxidized glutathione is one of the main substances that maintain redox homeostasis in the body. It has been reported that DBD pretreatment could enhance the glutathione status of blood cells, thereby improving their resistance to oxidative stress induced damage (Mak et al., 2006). Jun, as the most widely studied member of the transcription factor AP-1 (activator protein-1) family, was involved in a variety of cell activities, such as tumorigenesis, survival, apoptosis, proliferation and histomorphogenesis (Meng and Xia, 2011). Cxcl2 was a potent neutrophil chemoattractant, and this chemokine was almost completely derived from neutrophils (Girbl et al., 2018). Furthermore, Cxcl2 was also a mast cell and macrophage chemokine, which controlled the early stage of neutrophil recruitment during tissue inflammation (De Filippo et al., 2013). Studies have shown that Cxcl2 impairs the function of BMSCs (bone marrow mesenchymal stem cells) and could be used as a serum marker to indicate the BMSCs dysfunctions (Bi et al., 2021). Egr1 was crucial to the quiescence and self-renewal of HSCs (Yang et al., 2022), which were characterized by its self-renewal potential. In addition, Egr1 deficient mice showed a dramatically increase in the steady-state level of dividing HSCs in bone marrow and a significant spontaneous mobilization of HSC into peripheral blood (Min et al., 2008). The qRT-PCR results showed that MDBD could regulate the expression disorder of these main differential genes to approximate normal values to alleviate leukopenia.

## 5 Conclusion

Despite our growing understanding of pharmacodynamics and functions of MDBD in relieving radiation-induced leukopenia, details of the mechanism of leukocytosis increase remains unclear due to the complexity of the mechanism of the traditional Chinese medicine formula. We used abundant pharmacodynamic data to prove the efficacy of the new prescription MDBD, which was first introduced in treating radiation-induced leukopenia. In addition, the integrated analysis of metabolomics and transcriptomics was conducted to preliminarily explore the protective effect of MDBD on radiation-induced leukopenia in mice through multi-pathways, mainly including steroid biosynthesis and IL-17 signaling pathway, among which the key genes were Jun, Cxcl2 and Egr1.

## Data Availability

The datasets presented in this study can be found in online repositories. The names of the repository/repositories and accession number(s) can be found below: NCBI BioProject (https://www.ncbi.nlm.nih.gov/bioproject/), PRJNA942083.

## Abbreviations

MDBD, modified Danggui Buxue decoction; DBD, Danggui Buxue decoction; ICT, icaritin; WBC, white blood cell; RBC, red blood cell; PLT, platelet; HGB, hemoglobin; GM-CSF, granulocyte-macrophage colony-stimulating factor; G-CSF, granulocyte colony-stimulating factor; IL-6, interleukin-6; TPO, thrombopoietin; BMNCs, bone marrow nuclear cells; HSCs, hematopoietic stem cells; VIP, variable importance in projection; PCA, principal component analysis; PLS-DA, partial least-squares-discriminant analysis; DEGs, different expression genes; KEGG, kyoto encyclopedia of genes and genomes; CFU, colony formation unit.

## References

[B1] Allen C. Her S. Jaffray D. A. (2017). Radiotherapy for cancer: Present and future. Adv. Drug Deliv. Rev. 109, 1–2. 10.1016/j.addr.2017.01.004 28189183

[B2] Amatya N. Garg A. V. Gaffen S. L. (2017). IL-17 signaling: The yin and the yang. Trends Immunol. 38, 310–322. 10.1016/j.it.2017.01.006 28254169 PMC5411326

[B3] Bayne L. J. Beatty G. L. Jhala N. Clark C. E. Rhim A. D. Stanger B. Z. (2012). Tumor-derived granulocyte-macrophage colony-stimulating factor regulates myeloid inflammation and T cell immunity in pancreatic cancer. Cancer Cell 21, 822–835. 10.1016/j.ccr.2012.04.025 22698406 PMC3575028

[B4] Becher B. Tugues S. Greter M. (2016). GM-CSF: From growth factor to central mediator of tissue inflammation. Immunity 45, 963–973. 10.1016/j.immuni.2016.10.026 27851925

[B5] Bi J. Li Q. Yang Z. Cai L. Lv T. Yang X. (2021). CXCL2 impairs functions of bone marrow mesenchymal stem cells and can serve as a serum marker in high-fat diet-fed rats. Front. Cell Dev. Biol. 9, 687942. 10.3389/fcell.2021.687942 34327200 PMC8315099

[B6] Brendolan A. Rosado M. M. Carsetti R. Selleri L. Dear T. N. (2007). Development and function of the mammalian spleen. Bioessays 29, 166–177. 10.1002/bies.20528 17226804

[B7] Citrin D. E. (2017). Recent developments in radiotherapy. N. Engl. J. Med. 377, 1065–1075. 10.1056/NEJMra1608986 28902591

[B8] Cordeiro Gomes A. Hara T. Lim V. Y. Herndler-Brandstetter D. Nevius E. Sugiyama T. (2016). Hematopoietic stem cell niches produce lineage-instructive signals to control multipotent progenitor differentiation. Immunity 45, 1219–1231. 10.1016/j.immuni.2016.11.004 27913094 PMC5538583

[B9] Dai J. Lu Y. Yu C. Keller J. M. Mizokami A. Zhang J. (2010). Reversal of chemotherapy-induced leukopenia using granulocyte macrophage colony-stimulating factor promotes bone metastasis that can be blocked with osteoclast inhibitors. Cancer Res. 70, 5014–5023. 10.1158/0008-5472.CAN-10-0100 20501834 PMC2888854

[B10] De Filippo K. Dudeck A. Hasenberg M. Nye E. Van Rooijen N. Hartmann K. (2013). Mast cell and macrophage chemokines CXCL1/CXCL2 control the early stage of neutrophil recruitment during tissue inflammation. Blood 121, 4930–4937. 10.1182/blood-2013-02-486217 23645836

[B11] Delaney G. Jacob S. Featherstone C. Barton M. (2005). The role of radiotherapy in cancer treatment: Estimating optimal utilization from a review of evidence-based clinical guidelines. Cancer 104, 1129–1137. 10.1002/cncr.21324 16080176

[B12] Dhagat U. Hercus T. R. Broughton S. E. Nero T. L. Cheung Tung Shing K. S. Barry E. F. (2018). The mechanism of GM-CSF inhibition by human GM-CSF auto-antibodies suggests novel therapeutic opportunities. MAbs 10, 1018–1029. 10.1080/19420862.2018.1494107 29969365 PMC6204844

[B13] Dong N. Zhang X. Wu D. Hu Z. Liu W. Deng S. (2022). Medication regularity of traditional Chinese medicine in the treatment of aplastic anemia based on data mining. Evid. Based Complement. Altern. Med. 2022, 1605359. 10.1155/2022/1605359 PMC9436587 36062179

[B14] Du R. Bei H. Jia L. Huang C. Chen Q. Tao C. (2020). Danggui Buxue Tang restores antibiotic-induced metabolic disorders by remodeling the gut microbiota. J. Ethnopharmacol. 259, 112953. 10.1016/j.jep.2020.112953 32407936

[B15] Franco P. Ragona R. Arcadipane F. Mistrangelo M. Cassoni P. Rondi N. (2017). Dosimetric predictors of acute hematologic toxicity during concurrent intensity-modulated radiotherapy and chemotherapy for anal cancer. Clin. Transl. Oncol. 19, 67–75. 10.1007/s12094-016-1504-2 27037814

[B16] Girbl T. Lenn T. Perez L. Rolas L. Barkaway A. Thiriot A. (2018). Distinct compartmentalization of the chemokines CXCL1 and CXCL2 and the atypical receptor ACKR1 determine discrete stages of neutrophil diapedesis. Immunity 49, 1062–1076.e6. 10.1016/j.immuni.2018.09.018 30446388 PMC6303217

[B17] Gong A. G. Lau K. M. Xu M. L. Lin H. Q. Dong T. T. Zheng K. Y. (2016). The estrogenic properties of Danggui Buxue Tang, a Chinese herbal decoction, are triggered predominantly by calycosin in MCF-7 cells. J. Ethnopharmacol. 189, 81–89. 10.1016/j.jep.2016.05.035 27196297

[B18] Huang P. (2019). A study on the effective constituents and mechanisms of qi-jing-sheng-Bai Granule in treating leukopenia in mice. Shanghai, China: Shanghai University of Traditional Chinese Medicine.

[B19] Jairam V. Lee V. Park H. S. Thomas C. R. Jr. Melnick E. R. Gross C. P. (2019). Treatment-related complications of systemic therapy and radiotherapy. JAMA Oncol. 5, 1028–1035. 10.1001/jamaoncol.2019.0086 30946433 PMC6583836

[B20] Jia W. Xie G. Jia W. (2018). Bile acid-microbiota crosstalk in gastrointestinal inflammation and carcinogenesis. Nat. Rev. Gastroenterol. Hepatol. 15, 111–128. 10.1038/nrgastro.2017.119 29018272 PMC5899973

[B21] Kolls J. K. Linden A. (2004). Interleukin-17 family members and inflammation. Immunity 21, 467–476. 10.1016/j.immuni.2004.08.018 15485625

[B22] Koukourakis M. I. Giatromanolaki A. (2021). Lymphopenia and intratumoral lymphocytic balance in the era of cancer immuno-radiotherapy. Crit. Rev. Oncol. Hematol. 159, 103226. 10.1016/j.critrevonc.2021.103226 33482348

[B23] Kuo P. Bratman S. V. Shultz D. B. Von Eyben R. Chan C. Wang Z. (2014). Galectin-1 mediates radiation-related lymphopenia and attenuates NSCLC radiation response. Clin. Cancer Res. 20, 5558–5569. 10.1158/1078-0432.CCR-14-1138 25189484 PMC4216761

[B24] Kwan K. K. L. Huang Y. Leung K. W. Dong T. T. X. Tsim K. W. K. (2019). Danggui buxue tang, a Chinese herbal decoction containing astragali radix and angelicae sinensis radix, Modulates mitochondrial Bioenergetics in cultured cardiomyoblasts. Front. Pharmacol. 10, 614. 10.3389/fphar.2019.00614 31316376 PMC6611430

[B25] Lin H. Q. Gong A. G. Wang H. Y. Duan R. Dong T. T. Zhao K. J. (2017). Danggui buxue tang (astragali radix and angelicae sinensis radix) for menopausal symptoms: A review. J. Ethnopharmacol. 199, 205–210. 10.1016/j.jep.2017.01.044 28163116

[B26] Liu C. Li J. Meng F. Y. Liang S. X. Deng R. Li C. K. (2010). Polysaccharides from the root of Angelica sinensis promotes hematopoiesis and thrombopoiesis through the PI3K/AKT pathway. BMC Complement. Altern. Med. 10, 79. 10.1186/1472-6882-10-79 21176128 PMC3022894

[B27] Liu J. Huang C. Luo X. Xu J. (2008). Study on the establishment of mouse leukopenia model by ^60^Co irradiation. Pharmacol. Clin. Chin. Mater Med. 24, 65–70. 10.1007/978-3-211-78205-7_11

[B28] Liu Y. Chang M. Hu Z. Xu X. Wu W. Ning M. (2021). Danggui Buxue Decoction enhances the anticancer activity of gemcitabine and alleviates gemcitabine-induced myelosuppression. J. Ethnopharmacol. 273, 113965. 10.1016/j.jep.2021.113965 33639205

[B29] Ma C. Zhang Y. Dou X. Liu L. Zhang W. Ye J. (2022). Combining multiple acquisition modes and computational data annotation for structural characterization in traditional Chinese medicine: Miao Nationality medicine Qijiao Shengbai Capsule as a case study. RSC Adv. 12, 27781–27792. 10.1039/d2ra04720a 36320242 PMC9520537

[B30] Mak D. H. Chiu P. Y. Dong T. T. Tsim K. W. Ko K. M. (2006). Dang-Gui Buxue Tang produces a more potent cardioprotective effect than its component herb extracts and enhances glutathione status in rat heart mitochondria and erythrocytes. Phytother. Res. 20, 561–567. 10.1002/ptr.1904 16619337

[B31] Mari M. Morales A. Colell A. Garcia-Ruiz C. Fernandez-Checa J. C. (2009). Mitochondrial glutathione, a key survival antioxidant. Antioxid. Redox Signal 11, 2685–2700. 10.1089/ARS.2009.2695 19558212 PMC2821140

[B32] Meng Q. Xia Y. (2011). c-Jun, at the crossroad of the signaling network. Protein Cell 2, 889–898. 10.1007/s13238-011-1113-3 22180088 PMC4875184

[B33] Min I. M. Pietramaggiori G. Kim F. S. Passegue E. Stevenson K. E. Wagers A. J. (2008). The transcription factor EGR1 controls both the proliferation and localization of hematopoietic stem cells. Cell Stem Cell 2, 380–391. 10.1016/j.stem.2008.01.015 18397757

[B34] Nakae S. Nambu A. Sudo K. Iwakura Y. (2003). Suppression of immune induction of collagen-induced arthritis in IL-17-deficient mice. J. Immunol. 171, 6173–6177. 10.4049/jimmunol.171.11.6173 14634133

[B35] O'hagan-Wong K. Nadeau S. Carrier-Leclerc A. Apablaza F. Hamdy R. Shum-Tim D. (2016). Increased IL-6 secretion by aged human mesenchymal stromal cells disrupts hematopoietic stem and progenitor cells' homeostasis. Oncotarget 7, 13285–13296. 10.18632/oncotarget.7690 26934440 PMC4924641

[B36] Onishi R. M. Gaffen S. L. (2010). Interleukin-17 and its target genes: Mechanisms of interleukin-17 function in disease. Immunology 129, 311–321. 10.1111/j.1365-2567.2009.03240.x 20409152 PMC2826676

[B37] Park H. Li Z. Yang X. O. Chang S. H. Nurieva R. Wang Y. H. (2005). A distinct lineage of CD4 T cells regulates tissue inflammation by producing interleukin 17. Nat. Immunol. 6, 1133–1141. 10.1038/ni1261 16200068 PMC1618871

[B38] Pouliliou S. E. Lialiaris T. S. Dimitriou T. Giatromanolaki A. Papazoglou D. Pappa A. (2015). Survival fraction at 2 Gy and γH2AX expression kinetics in peripheral blood lymphocytes from cancer patients: Relationship with acute radiation-induced toxicities. Int. J. Radiat. Oncol. Biol. Phys. 92, 667–674. 10.1016/j.ijrobp.2015.02.023 25892583

[B39] Qin T. Ren Z. Yi L. Liu X. Luo Y. Long Y. (2019). Immunological modulation effects of an acid Epimedium polysaccharide on immune response in chickens. Int. Immunopharmacol. 70, 56–66. 10.1016/j.intimp.2019.02.009 30785091

[B40] Schiffer L. Barnard L. Baranowski E. S. Gilligan L. C. Taylor A. E. Arlt W. (2019). Human steroid biosynthesis, metabolism and excretion are differentially reflected by serum and urine steroid metabolomes: A comprehensive review. J. Steroid Biochem. Mol. Biol. 194, 105439. 10.1016/j.jsbmb.2019.105439 31362062 PMC6857441

[B41] Shi C. Han W. Zhang M. Zang R. Du K. Li L. (2020a). Sulfated polymannuroguluronate TGC161 ameliorates leukopenia by inhibiting CD4(+) T cell apoptosis. Carbohydr. Polym. 247, 116728. 10.1016/j.carbpol.2020.116728 32829850 PMC7336955

[B42] Shi X. Q. Zhu Z. H. Yue S. J. Tang Y. P. Chen Y. Y. Pu Z. J. (2020b). Integration of organ metabolomics and proteomics in exploring the blood enriching mechanism of Danggui Buxue Decoction in hemorrhagic anemia rats. J. Ethnopharmacol. 261, 113000. 10.1016/j.jep.2020.113000 32663590

[B43] Sun C. Yang J. Pan L. Guo N. Li B. Yao J. (2018). Improvement of icaritin on hematopoietic function in cyclophosphamide-induced myelosuppression mice. Immunopharmacol. Immunotoxicol. 40, 25–34. 10.1080/08923973.2017.1392564 29077519

[B44] Tian S. Huang P. Gu Y. Yang J. Wu R. Zhao J. (2019). Systems biology analysis of the effect and mechanism of qi-jing-sheng-Bai Granule on leucopenia in mice. Front. Pharmacol. 10, 408. 10.3389/fphar.2019.00408 31105563 PMC6494967

[B45] Wang W. L. Sheu S. Y. Chen Y. S. Kao S. T. Fu Y. T. Kuo T. F. (2015). Enhanced bone tissue regeneration by porous gelatin composites loaded with the Chinese herbal decoction danggui buxue tang. PLoS One 10, e0131999. 10.1371/journal.pone.0131999 26126113 PMC4488343

[B46] Wang X. Bei H. Du R. Chen Q. Wu F. Chen J. (2020). Metabolomic analysis of serum reveals the potential effective ingredients and pathways of Danggui Buxue Tang in promoting erythropoiesis. Complement. Ther. Med. 48, 102247. 10.1016/j.ctim.2019.102247 31987250

[B47] Xie J. H. Chen Z. W. Pan Y. W. Luo D. M. Su Z. R. Chen H. M. (2016). Evaluation of safety of modified-Danggui Buxue Tang in rodents:immunological, toxicity and hormonal aspects. J. Ethnopharmacol. 183, 59–70. 10.1016/j.jep.2015.12.049 26732632

[B48] Yang M. Chan G. C. Deng R. Ng M. H. Cheng S. W. Lau C. P. (2009). An herbal decoction of Radix astragali and Radix angelicae sinensis promotes hematopoiesis and thrombopoiesis. J. Ethnopharmacol. 124, 87–97. 10.1016/j.jep.2009.04.007 19443149

[B49] Yang X. Huang C. G. Du S. Y. Yang S. P. Zhang X. Liu J. Y. (2014). Effect of Danggui Buxue Tang on immune-mediated aplastic anemia bone marrow proliferation mice. Phytomedicine 21, 640–646. 10.1016/j.phymed.2013.10.018 24290471

[B50] Yang Y. Kueh A. J. Grant Z. L. Abeysekera W. Garnham A. L. Wilcox S. (2022). The histone lysine acetyltransferase HBO1 (KAT7) regulates hematopoietic stem cell quiescence and self-renewal. Blood 139, 845–858. 10.1182/blood.2021013954 34724565

[B51] Ye P. Rodriguez F. H. Kanaly S. Stocking K. L. Schurr J. Schwarzenberger P. (2001). Requirement of interleukin 17 receptor signaling for lung CXC chemokine and granulocyte colony-stimulating factor expression, neutrophil recruitment, and host defense. J. Exp. Med. 194, 519–527. 10.1084/jem.194.4.519 11514607 PMC2193502

[B52] Zheng K. Y. Choi R. C. Xie H. Q. Cheung A. W. Guo A. J. Leung K. W. (2010). The expression of erythropoietin triggered by danggui buxue tang, a Chinese herbal decoction prepared from radix Astragali and radix Angelicae Sinensis, is mediated by the hypoxia-inducible factor in cultured HEK293T cells. J. Ethnopharmacol. 132, 259–267. 10.1016/j.jep.2010.08.029 20723591

