# Supplementary material for: Integrated transcriptomic and metabolomic profiles reveal the protective mechanism of modified Danggui Buxue decoction on radiation-induced leukopenia in mice
Source: Front Pharmacol. 2023 Aug 3;14:1178724. doi: 10.3389/fphar.2023.1178724 (PMC10434993; doi:10.3389/fphar.2023.1178724)
Supplement: Supplementary file 1 [file DataSheet1.docx]

Supplementary Material

# Supplementary Figures


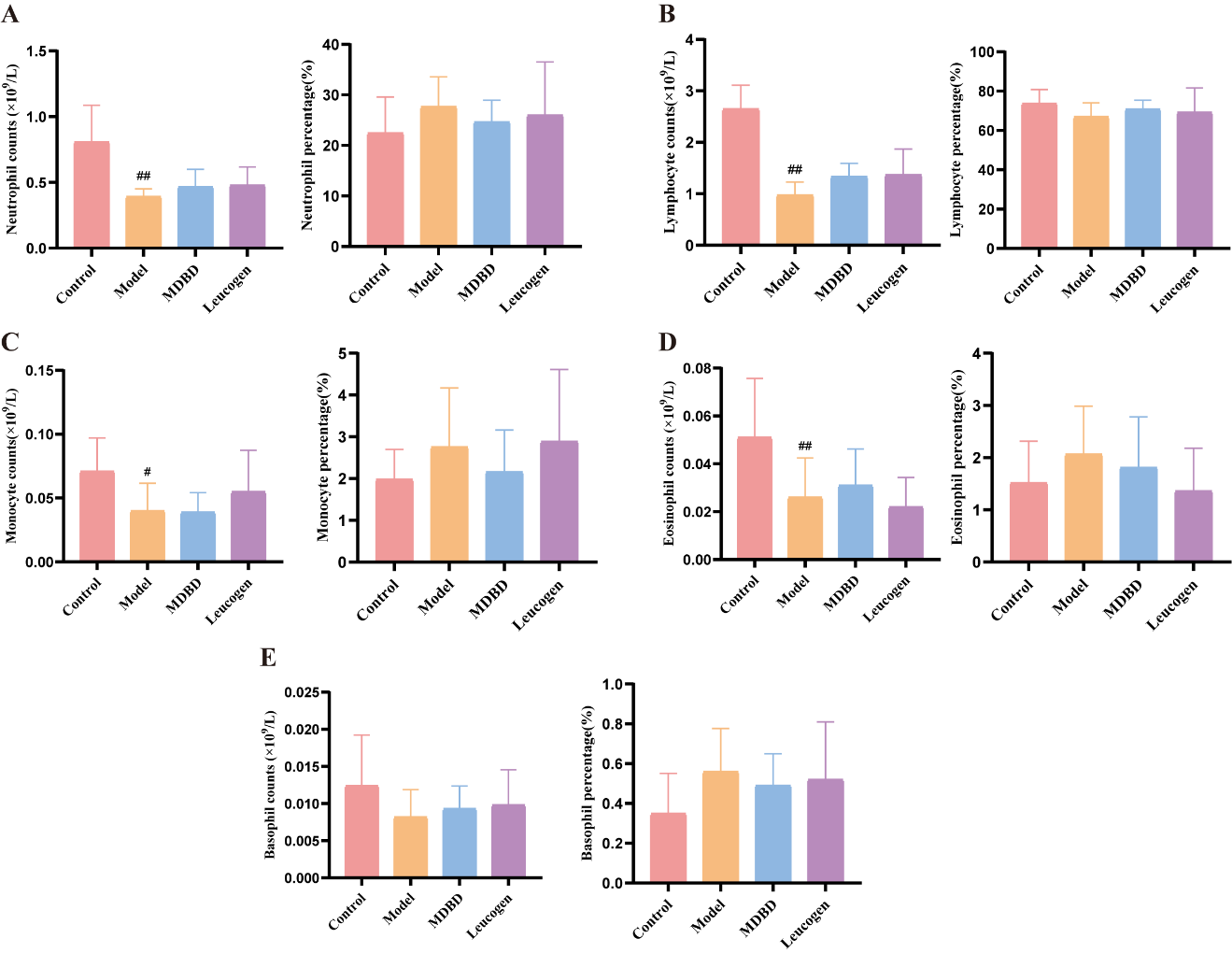


**Supplementary Figure S1.** The effect of MDBD on WBC differential counts: proportion and counts of neutrophil (A), proportion and counts of lymphocyte (B), proportion and counts of monocyte (C), proportion and counts of eosinophil (D), and proportion and counts of basophil (E) in leukopenia mice induced by radiation. Data are presented as means ± SD, #*p* < 0.05, ##*p* < 0.01 vs. control group.


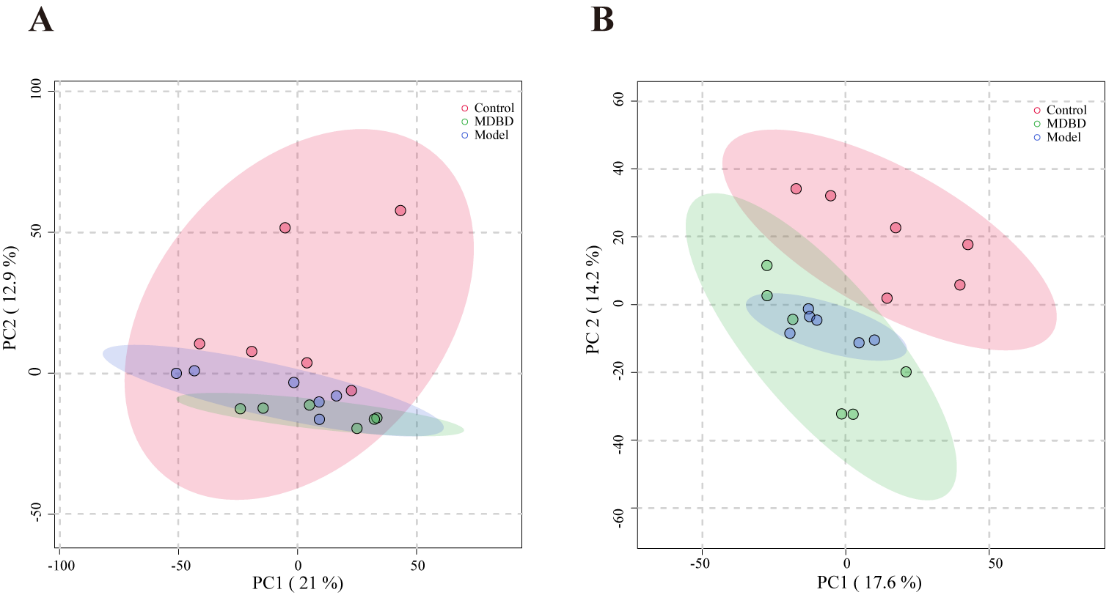


**Supplementary Figure S2.** Two-dimensional PCA score plots in ESI+(A) and ESI– mode (B).


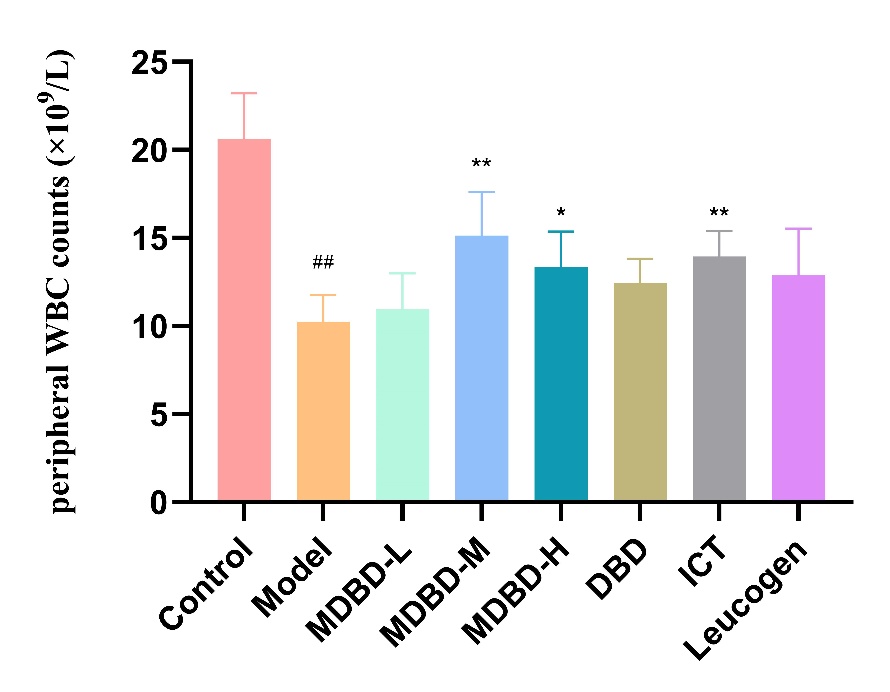
 **Supplementary Figure S3.** White blood cell counts of tail vein on the 14th day after radiation (n=7). The data are expressed as means ± SD. ##*p* < 0.01 *vs*. control group; ***p* < 0.01 *vs*. model group.


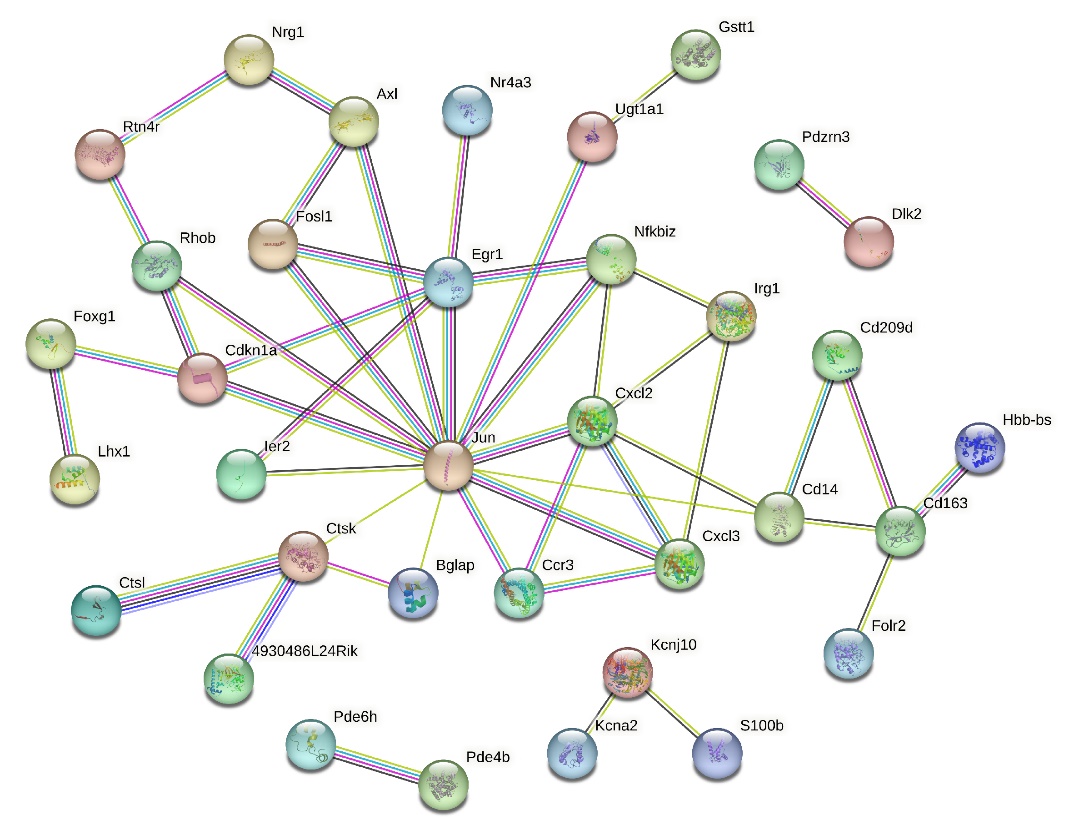


**Supplementary Figure S4.** PPI analysis of 158 DEGs.

# Supplementary Tables

**Table S1** Primer sequences used in the qRT-PCR analysis.

| Gene |  | **Sequence** |
| --- | --- | --- |
| Cxcl2 | Forward  Reverse | TCA ACG GAA GAA CCA AAG AGA  GTG AAC TCT CAG ACA GCG AGG |
| Egr1 | Forward  Reverse | GGA CAC GCT CAC CTT AGC CTTA  CTC AGC CCT CTT CCT CGT TTT |
| Jun | Forward  Reverse | AGC AAC TTT CCT GAC CCA GAG  TCT TTA CAG TCT CGG TGG CAG |
| β-actin | Forward  Reverse | CCT CTA TGC CAA CAC AGT  AGC CAC CAA TCC ACA CAG |

**Table S2** Potential biomarkers in serum associated with MDBD treatment based on LC/MS analysis in radiation-induced leukopenia mice.

| NO. | RT | Compound ID | Formula | *m/z* | Metabolite | Trend | |
| --- | --- | --- | --- | --- | --- | --- | --- |
|  |  |  |  |  |  | Model/Control | MDBD/Model |
| 1 | 0.681 | HMDB0003337 | C_20_H_32_N_6_O_12_S_2_ | 611.1430 | Oxidized glutathione | ↓** | ↑## |
| 2 | 2.132 | HMDB0013189 | C_15_H_15_NO_8_ | 336.0727 | 3-Indolecarboxylate glucuronide | ↓ | ↑# |
| 3 | 2.309 | HMDB0241087 | C_17_H_29_NO_7_ | 404.1912 | 5-Hydroxydec-6-enedioylcarnitine | ↓* | ↑ |
| 4 | 2.331 | HMDB0010328 | C_14_H_19_NO_7_ | 348.0860 | Tyramine glucuronide (beta-D-Glucuronide) | ↓** | ↑ |
| 5 | 2.707 | HMDB0060416 | C_10_H_13_N_4_O_7_PS | 363.0167 | 6-Thioinosine-5'-monophosphate | ↑* | ↓ |
| 6 | 2.899 | HMDB0062551 | C_8_H_10_O_4_S | 201.0216 | 4-Ethylphenylsulfate | ↑ | ↓# |
| 7 | 3.034 | HMDB0002639 | C_26_H_43_NO_7_S | 512.2675 | Sulfolithocholylglycine | ↓** | ↑ |
| 8 | 3.063 | HMDB0000792 | C_10_H_18_O_4_ | 201.1121 | Sebacic acid | ↓** | ↑ |
| 9 | 3.312 | HMDB0255727 | C_23_H_38_O_5_ | 393.2629 | Norcholic acid | ↓ | ↑# |
| 10 | 3.490 | HMDB0000619 | C_24_H_40_O_5_ | 407.2792 | Cholic acid | ↓* | ↑ |
| 11 | 3.598 | HMDB0001140 | C_8_H_16_O | 274.2764 | Octanal | ↓** | ↑ |
| 12 | 3.710 | HMDB0000874 | C_26_H_45_NO_6_S | 498.2880 | Tauroursodeoxycholic acid | ↓** | ↑ |
| 13 | 3.853 | HMDB0001085 | C_20_H_32_O_4_ | 371.2067 | Leukotriene B4 | ↓** | ↑# |
| 14 | 3.902 | HMDB0000391 | C_24_H_38_O_5_ | 405.2632 | 7-Ketodeoxycholic acid | ↓* | ↑ |
| 15 | 3.923 | HMDB0012558 | C_28_H_40_O_4_ | 475.2670 | 13'-Carboxy-γ-tocotrienol | ↓* | ↑ |
| 16 | 3.923 | HMDB0000917 | C_24_H_40_O_5_ | 407.2793 | Ursocholic acid | ↓* | ↑ |
| 17 | 3.968 | HMDB0002014 | C_21_H_39_NO_4_ | 370.2990 | cis-5-Tetradecenoylcarnitine | ↓* | ↑ |
| 18 | 4.153 | HMDB0005066 | C_21_H_41_NO_4_ | 372.3141 | Tetradecanoylcarnitine | ↓* | ↑ |
| 19 | 4.187 | HMDB0012503 | C_20_H_34_O_7_ | 385.2222 | 10,11-dihydro-20-trihydroxy-leukotriene B4 | ↓** | ↑ |
| 20 | 4.189 | HMDB0000067 | C_27_H_46_O | 369.3541 | Cholesterol (Cholest-5-en-3beta-ol) | ↓ | ↑# |
| 21 | 4.229 | HMDB0012562 | C_28_H_42_O_3_ | 471.3109 | 13'-Hydroxy-γ-tocotrienol | ↓** | ↑ |
| 22 | 4.572 | HMDB0000222 | C_23_H_45_NO_4_ | 400.3455 | Palmitoylcarnitine | ↓** | ↑ |
| 23 | 4.755 | HMDB0000946 | C_24_H_40_O_4_ | 391.2840 | Ursodeoxycholic acid | ↓** | ↑# |
| 24 | 4.786 | HMDB0006841 | C_27_H_46_O | 369.3540 | 5alpha-Cholest-8-en-3beta-ol | ↓ | ↑# |
| 25 | 5.192 | HMDB0001170 | C_27_H_46_O | 369.3539 | Lathosterol | ↓ | ↑# |
| 26 | 5.445 | HMDB0010734 | C_16_H_32_O_3_ | 271.2273 | (R)-3-Hydroxy-hexadecanoic acid | ↑* | ↓# |
| 27 | 6.213 | HMDB0001256 | C_10_H_26_N_4_ | 237.1853 | Spermine | ↓** | ↑ |
| 28 | 6.213 | HMDB0032054 | C_15_H_26_O_2_ | 237.1853 | alpha-Terpinyl pentanoate | ↓** | ↑ |
| 29 | 10.039 | HMDB0036283 | C_37_H_54_O_3_ | 591.3924 | Campesteryl p-coumaric acid | ↑ | ↓# |
| 30 | 10.416 | HMDB0031288 | C_21_H_44_O_2_ | 327.3271 | 1,21-Heneicosanediol | ↓* | ↑ |
| 31 | 12.250 | HMDB0011512 | C_25_H_50_NO_7_P | 506.3239 | LysoPE(20:1(11Z)/0:0) | ↓ | ↑ |
| 32 | 12.947 | HMDB0011527 | C_29_H_60_NO_7_P | 564.4027 | LysoPE(24:0/0:0) | ↑** | ↓ |
| 33 | 13.204 | HMDB0265620 | C_45_H_79_O_11_P | 871.5394 | PA(22:0/20:5(7Z,9Z,11E,13E,17Z)-3OH(5,6,15)) | ↑ | ↓# |
| 34 | 13.204 | HMDB0265678 | C_45_H_79_O_11_P | 871.5394 | PA(22:1(13Z)/PGE2) | ↑ | ↓# |
| 35 | 13.527 | HMDB0012273 | C_16_H_33_NO | 256.2642 | Palmitic amide | ↑** | ↓## |

Note: ↑, increase; ↓, and decrease; Model/Control: Model versus Control; MDBD/Model: MDBD versus Model; **p* < 0.05, ***p* < 0.01 vs. control group; #*p* < 0.05, ##*p* < 0.01 vs. model group.

**Table S3** DEGs regulated by MDBD in radiation-induced leukopenia mice.

| NO. | Gene ID | Gene name | Model/Control | MDBD/Model |
| --- | --- | --- | --- | --- |
| 1 | ENSMUSG00000053219 | Raet1e | yes\|down | yes\|up |
| 2 | ENSMUSG00000028341 | Nr4a3 | yes\|up | yes\|down |
| 3 | ENSMUSG00000053581 | Zfand2a | yes\|up | yes\|down |
| 4 | ENSMUSG00000087026 | A230103J11Rik | yes\|down | yes\|up |
| 5 | ENSMUSG00000095338 | Igkv3-9 | yes\|up | yes\|down |
| 6 | ENSMUSG00000078866 | Zfp970 | yes\|down | yes\|up |
| 7 | ENSMUSG00000086424 | Gm15569 | yes\|up | yes\|down |
| 8 | ENSMUSG00000114711 | Gm47759 | yes\|up | yes\|down |
| 9 | ENSMUSG00000119132 | Gm24407 | yes\|up | yes\|down |
| 10 | ENSMUSG00000022126 | Acod1 | yes\|up | yes\|down |
| 11 | ENSMUSG00000105906 | Iglc1 | yes\|up | yes\|up |
| 12 | ENSMUSG00000006014 | Prg4 | yes\|down | yes\|up |
| 13 | ENSMUSG00000018698 | Lhx1 | yes\|down | yes\|down |
| 14 | ENSMUSG00000106609 | Gm43181 | yes\|up | yes\|down |
| 15 | ENSMUSG00000064330 | Pde6h | yes\|up | yes\|down |
| 16 | ENSMUSG00000103144 | Pcdhga1 | yes\|down | yes\|up |
| 17 | ENSMUSG00000100969 | 1700030N03Rik | yes\|up | yes\|down |
| 18 | ENSMUSG00000030651 | Art2b | yes\|down | yes\|down |
| 19 | ENSMUSG00000052392 | Acot4 | yes\|up | yes\|down |
| 20 | ENSMUSG00000104467 | Gm37660 | yes\|up | yes\|down |
| 21 | ENSMUSG00000084329 | Gm6733 | yes\|up | yes\|down |
| 22 | ENSMUSG00000057802 | Gm10030 | yes\|up | yes\|down |
| 23 | ENSMUSG00000081742 | Gm9051 | yes\|up | yes\|down |
| 24 | ENSMUSG00000094338 | H2bc13 | yes\|up | yes\|down |
| 25 | ENSMUSG00000028525 | Pde4b | yes\|up | yes\|down |
| 26 | ENSMUSG00000035448 | Ccr3 | yes\|down | yes\|up |
| 27 | ENSMUSG00000006378 | Gcat | yes\|down | yes\|up |
| 28 | ENSMUSG00000103558 | Gm38220 | yes\|up | yes\|down |
| 29 | ENSMUSG00000096594 | Igkv8-19 | yes\|up | yes\|down |
| 30 | ENSMUSG00000038418 | Egr1 | yes\|up | yes\|down |
| 31 | ENSMUSG00000037762 | Slc16a9 | yes\|down | yes\|up |
| 32 | ENSMUSG00000033208 | S100b | yes\|up | yes\|down |
| 33 | ENSMUSG00000067147 | Rpl7a-ps11 | yes\|down | yes\|up |
| 34 | ENSMUSG00000097576 | D930030I03Rik | yes\|up | yes\|down |
| 35 | ENSMUSG00000112505 | Gm48610 | yes\|up | yes\|down |
| 36 | ENSMUSG00000076556 | Igkv4-57 | yes\|down | yes\|up |
| 37 | ENSMUSG00000113817 | Gm21056 | yes\|up | yes\|down |
| 38 | ENSMUSG00000066235 | Pomgnt2 | yes\|down | yes\|up |
| 39 | ENSMUSG00000022658 | Tagln3 | yes\|down | yes\|up |
| 40 | ENSMUSG00000039349 | C130074G19Rik | yes\|up | yes\|down |
| 41 | ENSMUSG00000043668 | Tox3 | yes\|down | yes\|up |
| 42 | ENSMUSG00000032076 | Cadm1 | yes\|down | yes\|up |
| 43 | ENSMUSG00000020656 | Grhl1 | yes\|up | yes\|down |
| 44 | ENSMUSG00000109719 | Gm45266 | yes\|up | yes\|down |
| 45 | ENSMUSG00000099647 | Gm5776 | yes\|down | yes\|up |
| 46 | ENSMUSG00000097365 | C030034L19Rik | yes\|down | yes\|up |
| 47 | ENSMUSG00000058626 | Capn11 | yes\|up | yes\|down |
| 48 | ENSMUSG00000031342 | Gpm6b | yes\|down | yes\|up |
| 49 | ENSMUSG00000023067 | Cdkn1a | yes\|up | yes\|down |
| 50 | ENSMUSG00000040297 | Suco | yes\|up | yes\|down |
| 51 | ENSMUSG00000074483 | Bglap | yes\|up | yes\|down |
| 52 | ENSMUSG00000102151 | Gm37472 | yes\|up | yes\|down |
| 53 | ENSMUSG00000030178 | Klra13-ps | yes\|down | yes\|down |
| 54 | ENSMUSG00000090027 | Gm15740 | yes\|up | yes\|down |
| 55 | ENSMUSG00000090026 | Gm15996 | yes\|down | yes\|up |
| 56 | ENSMUSG00000115210 | Gm49308 | yes\|up | yes\|down |
| 57 | ENSMUSG00000105296 | Gm19708 | yes\|down | yes\|up |
| 58 | ENSMUSG00000035356 | Nfkbiz | yes\|up | yes\|down |
| 59 | ENSMUSG00000089960 | Ugt1a1 | yes\|down | yes\|up |
| 60 | ENSMUSG00000107846 | Gm43963 | yes\|up | yes\|down |
| 61 | ENSMUSG00000103103 | 4833445I07Rik | yes\|up | yes\|down |
| 62 | ENSMUSG00000020067 | Mypn | yes\|down | yes\|up |
| 63 | ENSMUSG00000058427 | Cxcl2 | yes\|up | yes\|down |
| 64 | ENSMUSG00000031495 | Cd209d | yes\|down | yes\|up |
| 65 | ENSMUSG00000094799 | No matches | yes\|up | yes\|down |
| 66 | ENSMUSG00000032725 | Folr2 | yes\|down | yes\|up |
| 67 | ENSMUSG00000097842 | 9330104G04Rik | yes\|down | yes\|up |
| 68 | ENSMUSG00000074108 | Rpl10-ps2 | yes\|up | yes\|down |
| 69 | ENSMUSG00000106696 | Gm42729 | yes\|up | yes\|down |
| 70 | ENSMUSG00000028457 | Atp8b5 | yes\|down | yes\|up |
| 71 | ENSMUSG00000117440 | Gm50087 | yes\|up | yes\|down |
| 72 | ENSMUSG00000054364 | Rhob | yes\|up | yes\|down |
| 73 | ENSMUSG00000043811 | Rtn4r | yes\|down | yes\|up |
| 74 | ENSMUSG00000097207 | 6030443J06Rik | yes\|down | yes\|up |
| 75 | ENSMUSG00000024912 | Fosl1 | yes\|up | yes\|down |
| 76 | ENSMUSG00000111329 | A830035O19Rik | yes\|up | yes\|down |
| 77 | ENSMUSG00000021477 | Ctsl | yes\|up | yes\|down |
| 78 | ENSMUSG00000051022 | Hs3st1 | yes\|down | yes\|down |
| 79 | ENSMUSG00000080538 | Gm25541 | yes\|up | yes\|down |
| 80 | ENSMUSG00000028111 | Ctsk | yes\|down | yes\|up |
| 81 | ENSMUSG00000008845 | Cd163 | yes\|down | yes\|up |
| 82 | ENSMUSG00000100302 | Gm29670 | yes\|up | yes\|down |
| 83 | ENSMUSG00000113792 | Gm10933 | yes\|down | yes\|up |
| 84 | ENSMUSG00000018924 | Alox15 | yes\|down | yes\|up |
| 85 | ENSMUSG00000006411 | Nectin4 | yes\|down | yes\|up |
| 86 | ENSMUSG00000031613 | Hpgd | yes\|down | yes\|up |
| 87 | ENSMUSG00000085720 | Gm7854 | yes\|up | yes\|down |
| 88 | ENSMUSG00000074063 | Osgin1 | yes\|up | yes\|down |
| 89 | ENSMUSG00000046733 | Gprc5a | yes\|up | yes\|down |
| 90 | ENSMUSG00000068141 | Gm10232 | yes\|up | yes\|down |
| 91 | ENSMUSG00000113094 | Gm47447 | yes\|up | yes\|down |
| 92 | ENSMUSG00000021879 | Dnah12 | yes\|down | yes\|up |
| 93 | ENSMUSG00000041633 | Kctd12b | yes\|down | yes\|up |
| 94 | ENSMUSG00000004359 | Spic | yes\|down | yes\|up |
| 95 | ENSMUSG00000031266 | Gla | yes\|up | yes\|down |
| 96 | ENSMUSG00000047293 | Gpr15 | yes\|down | yes\|up |
| 97 | ENSMUSG00000052684 | Jun | yes\|up | yes\|down |
| 98 | ENSMUSG00000114865 | Gm29776 | yes\|up | yes\|down |
| 99 | ENSMUSG00002076763 | No matches | yes\|up | yes\|down |
| 100 | ENSMUSG00000046727 | Cystm1 | yes\|up | yes\|down |
| 101 | ENSMUSG00000002602 | Axl | yes\|down | yes\|up |
| 102 | ENSMUSG00000105881 | 4932422M17Rik | yes\|down | yes\|up |
| 103 | ENSMUSG00000073787 | Gm10575 | yes\|down | yes\|up |
| 104 | ENSMUSG00000043102 | Qrfp | yes\|down | yes\|up |
| 105 | ENSMUSG00000106031 | Gm8848 | yes\|up | yes\|up |
| 106 | ENSMUSG00000066705 | Fxyd6 | yes\|down | yes\|down |
| 107 | ENSMUSG00000035357 | Pdzrn3 | yes\|up | yes\|down |
| 108 | ENSMUSG00000057135 | Scimp | yes\|down | yes\|up |
| 109 | ENSMUSG00000078151 | Gm12226 | yes\|up | yes\|down |
| 110 | ENSMUSG00000106149 | Gm43430 | yes\|up | yes\|down |
| 111 | ENSMUSG00000087064 | Sap30bpos | yes\|up | yes\|down |
| 112 | ENSMUSG00000052305 | Hbb-bs | yes\|up | yes\|up |
| 113 | ENSMUSG00000052675 | Zfp112 | yes\|up | yes\|down |
| 114 | ENSMUSG00000112860 | Gm47439 | yes\|up | yes\|down |
| 115 | ENSMUSG00000053560 | Ier2 | yes\|up | yes\|down |
| 116 | ENSMUSG00000107143 | Gm6598 | yes\|up | yes\|down |
| 117 | ENSMUSG00000117534 | Gm20161 | yes\|down | yes\|up |
| 118 | ENSMUSG00000101751 | Gm2427 | yes\|up | yes\|down |
| 119 | ENSMUSG00000098609 | Anxa11os | yes\|up | yes\|down |
| 120 | ENSMUSG00000105432 | Gm43218 | yes\|up | yes\|up |
| 121 | ENSMUSG00000049709 | Nlrp10 | yes\|down | yes\|up |
| 122 | ENSMUSG00000110350 | Gm10252 | yes\|up | yes\|down |
| 123 | ENSMUSG00000047940 | Stpg2 | yes\|up | yes\|down |
| 124 | ENSMUSG00000042087 | 4933440N22Rik | yes\|up | yes\|down |
| 125 | ENSMUSG00000087055 | Gm11948 | yes\|up | yes\|down |
| 126 | ENSMUSG00000044708 | Kcnj10 | yes\|down | yes\|up |
| 127 | ENSMUSG00000096078 | Ighv1-62-2 | yes\|up | yes\|up |
| 128 | ENSMUSG00000050345 | 4930486L24Rik | yes\|down | yes\|up |
| 129 | ENSMUSG00000048814 | Lonrf2 | yes\|down | yes\|up |
| 130 | ENSMUSG00000020950 | Foxg1 | yes\|up | yes\|down |
| 131 | ENSMUSG00000095204 | Ighv1-52 | yes\|up | yes\|down |
| 132 | ENSMUSG00000021200 | Asb2 | yes\|down | yes\|up |
| 133 | ENSMUSG00000105201 | Gm43362 | yes\|up | yes\|down |
| 134 | ENSMUSG00000072612 | Gm10382 | yes\|up | yes\|down |
| 135 | ENSMUSG00000047428 | Dlk2 | yes\|up | yes\|down |
| 136 | ENSMUSG00000028784 | Spocd1 | yes\|down | yes\|up |
| 137 | ENSMUSG00000034205 | Loxl2 | yes\|up | yes\|down |
| 138 | ENSMUSG00000062991 | Nrg1 | yes\|down | yes\|up |
| 139 | ENSMUSG00000073728 | Tmem51os1 | yes\|down | yes\|up |
| 140 | ENSMUSG00000079710 | Dynlt2a2 | yes\|up | yes\|down |
| 141 | ENSMUSG00000090628 | Gm17083 | yes\|up | yes\|down |
| 142 | ENSMUSG00000027316 | Gfra4 | yes\|down | yes\|up |
| 143 | ENSMUSG00000072601 | Ear1 | yes\|down | yes\|up |
| 144 | ENSMUSG00000094652 | Ighv1-42 | yes\|down | yes\|up |
| 145 | ENSMUSG00000040724 | Kcna2 | yes\|down | yes\|up |
| 146 | ENSMUSG00000029379 | Cxcl3 | yes\|up | yes\|down |
| 147 | ENSMUSG00000020893 | Per1 | yes\|up | yes\|down |
| 148 | ENSMUSG00000043629 | 1700019D03Rik | yes\|up | yes\|down |
| 149 | ENSMUSG00000039246 | Lyplal1 | yes\|down | yes\|up |
| 150 | ENSMUSG00000082454 | Gm12183 | yes\|up | yes\|down |
| 151 | ENSMUSG00000053930 | Shisa6 | yes\|up | yes\|down |
| 152 | ENSMUSG00000078875 | Gm14419 | yes\|down | yes\|up |
| 153 | ENSMUSG00000001663 | Gstt1 | yes\|down | yes\|up |
| 154 | ENSMUSG00000020205 | Phlda1 | yes\|up | yes\|down |
| 155 | ENSMUSG00000057605 | Gm6807 | yes\|up | yes\|down |
| 156 | ENSMUSG00000097296 | Gm26532 | yes\|down | yes\|up |
| 157 | ENSMUSG00000110537 | Gm4316 | yes\|up | yes\|down |
| 158 | ENSMUSG00000051439 | Cd14 | yes\|up | yes\|down |

Note: yes|up: significant difference and up-regulation; yes|down: significant difference and down-regulation; No matches: No matching gene name; Model/Control: Model versus Control; MDBD/Model: MDBD versus Model.
